# Carbon source–sink relationship in *Arabidopsis thaliana*: the role of sucrose transporters

**DOI:** 10.1007/s00425-017-2807-4

**Published:** 2017-11-14

**Authors:** Mickaël Durand, Dany Mainson, Benoît Porcheron, Laurence Maurousset, Rémi Lemoine, Nathalie Pourtau

**Affiliations:** Université de Poitiers, UMR CNRS 7267 EBI Ecologie et Biologie des Interactions, Equipe “Sucres & Echanges Végétaux-Environnement”, Bâtiment B31, 3 rue Jacques Fort, TSA 51106, 86073 Poitiers Cedex 9, France

**Keywords:** Diel cycle, Full life development, Hydroponic culture, Osmotic stress, Roots, Sugar transporter gene expression

## Abstract

**The regulation of source-to-sink sucrose transport is associated with** ***AtSUC*** **and** ***AtSWEET*** **sucrose transporters’ gene expression changes in plants grown hydroponically under different physiological conditions.**

Source-to-sink transport of sucrose is one of the major determinants of plant growth. Whole-plant carbohydrates’ partitioning requires the specific activity of membrane sugar transporters. In *Arabidopsis thaliana* plants, two families of transporters are involved in sucrose transport: AtSUCs and AtSWEETs. This study is focused on the comparison of sucrose transporter gene expression, soluble sugar and starch levels and long distance sucrose transport, in leaves and sink organs (mainly roots) in different physiological conditions (along the plant life cycle, during a diel cycle, and during an osmotic stress) in plants grown hydroponically. In leaves, the *AtSUC2*, *AtSWEET11,* and *12* genes known to be involved in phloem loading were highly expressed when sucrose export was high and reduced during osmotic stress. In roots, *AtSUC1* was highly expressed and its expression profile in the different conditions tested suggests that it may play a role in sucrose unloading in roots and in root growth. The *SWEET* transporter genes *AtSWEET12, 13,* and *15* were found expressed in all organs at all stages studied, while differential expression was noticed for *AtSWEET14* in roots, stems, and siliques and *AtSWEET9, 10* expressions were only detected in stems and siliques. A role for these transporters in carbohydrate partitioning in different source–sink status is proposed, with a specific attention on carbon demand in roots. During development, despite trophic competition with others sinks, roots remained a significant sink, but during osmotic stress, the amount of translocated [U-^14^C]-sucrose decreased for rosettes and roots. Altogether, these results suggest that source–sink relationship may be linked with the regulation of sucrose transporter gene expression.

**Electronic supplementary material:**

The online version of this article (10.1007/s00425-017-2807-4) contains supplementary material, which is available to authorized users.

## Introduction

Plants grow autotrophically and perform photosynthesis by assimilating CO_2_ and using light and water to produce carbohydrates. Photosynthates make carbon (C) available for growth and maintenance of non-photosynthetic organs. C fixation occurs in source organs such as mature leaves that are photosynthetically active tissues. C fixed in excess during the day can be exported as sucrose to the non-photosynthetic sink organs such as roots, young leaves, reproductive organs, seeds, tubers and meristems (Koch 2004). However, assimilated C can also be transiently stored as starch in chloroplasts or as sucrose in the vacuole and then remobilised when sink demand exceeds photosynthetic C supply, as for example during the night (Smith and Stitt 2007; Graf and Smith 2011).

Because up to 80% of fixed C is exported to sinks (Kalt-Torres and Huber 1987), regulation of C partitioning is vital for plant growth and development (Gifford et al. 1984). The amount of C available depends on the type of organs, their state of development and environmental conditions (Stitt 1991; Paul and Foyer 2001). Source-to sink transport of sugar is one of the major determinants of plant growth and implicates a fine-tuned regulation of carbon allocation across plant organs through the phloem (Lemoine et al. 2013). During its life cycle, a plant has to face changes in C allocation linked with the competition status among various sinks for sugar availability. During vegetative stages, root and young leaves are major sinks, whereas fruits and seeds become dominant sinks during reproductive stages (Wardlaw 1990). At the end of development it can be assumed that two sinks, roots and seeds, enter in competition for carbon resources and fluxes will be determined by their respective “sink strength” (Ho 1988). At this point, roots are still consuming energy to provide enough water and mineral nutrients to complete whole-plant development, particularly seeds filling.

Although it is generally assumed that growth and development in plant is an integrated process in which primary assimilation in source tissues is balanced by the metabolic needs of heterotrophic sinks, the control of photosynthate partitioning is not so well understood but likely involves a complex regulatory network dependant on plant age and species (Afoufa-Bastien et al. 2010). Environmental factors are also known to impact source–sink relationship and can affect C allocation and plant growth (Lemoine et al. 2013). In the future, water availability is predicted to decrease dramatically in several areas of the world with large impacts on crop productivity. As a result of water deficit, photosynthesis decreases (Cornic 2000) and has an impact on the amount of carbon translocated to sink organs, especially roots (Hummel et al. 2010; Durand et al. 2016). Screening for root traits involved in drought tolerance appears as one of the promising research area to develop crop cultivars adapted to limited water availability (Ali et al. 2016). Therefore, understanding the C source–sink relationships to roots is a major goal for breeders. In leaves of plants subjected to drought, carbohydrates level increases as a consequence of growth leaf limitation. Excess of C could be stored as starch or kept as soluble carbohydrates, both participating to increase availability of C for the roots (Hummel et al. 2010). Sugar accumulation might also play a role in osmotic adjustment to maintain metabolic activity in source leaves (Chen and Jiang 2010).

As in many plants, in *Arabidopsis thaliana*, the major photosynthetic compound exported from source leaves is sucrose and it is allocated to heterotrophic sink tissue through the phloem (Lemoine 2000). Sucrose produced in mesophyll cells of mature leaves moves symplastically through plasmodesmata to the phloem parenchyma (PP) cells closed to collection phloem cells. In apoplastic loading species as *A. thaliana*, sucrose is actively loaded from the apoplast into the companion cell-sieve element (CC/SE) complex (Haritatos et al. 2000). Apoplastic phloem loading requires proteins to allow efficient sucrose movement across membranes. The release of sucrose into the apoplast is supposed to be facilitated by members of the SWEETs transporters family (AtSWEET11 and AtSWEET12) (Chen et al. 2012) and sucrose is then accumulated in the companion cell (CC) by an energy dependent AtSUC2 H^+^/sucrose symporter (Stadler and Sauer 1996; Gottwald et al. 2000) and finally passes to the sieve element (SE) through plasmodesmata. The high concentration of sucrose in the SE increases turgor pressure allowing sucrose transport in the sieve tubes to the sink organ according to the Mass-Flow model (Münch 1930). Unloading of sucrose from the phloem can occur through symplastic or apoplastic pathways depending on tissue types or development stages, but always where the turgor pressure drops. Apoplastic sucrose unloading involves sucrose transporters in sink organs or its conversion to hexoses by a cell-wall invertase (Ayre 2011; Ludewig and Flügge 2013; Ruan 2014).

The expression of many sucrose transporters genes has been reported in the roots of *Arabidopsis*, such as *SUC2* (Truernit and Sauer 1995; Stadler and Sauer 1996), *SUC1* (Sivitz et al. 2007, 2008), *SUC3* (Meyer et al. 2004), *SUC4* (Schneider et al. 2012) and *SUC5* (Baud et al. 2005), but their exact role in roots, especially for SUC2, has not been completely unveiled. Moreover, many data on *Arabidopsis* were obtained from young seedlings grown in agar plates and therefore concerned the early stages of development only.

To gain more information about source-to-sink transport regulation with emphasis on roots, we have settled an experimental system allowing the rapid and easy collection of the integral root systems. Plants were grown hydroponically in an Araponics^®^ growing system. The expression of sucrose transporter genes was monitored under several conditions with different source/sink relations. We first checked that transporter gene expression was not too dependent on the day–night cycle, so that a convenient time for harvest was chosen. The same analysis was conducted in two contrasting situations: during the whole development cycle with four compartments (rosette, roots, stems and siliques), and in response to osmotic stress with two compartments (rosette and roots). Our results shed light on a significant role played by the sucrose transporters SUC2 and SWEET11 and 12 in phloem loading and demonstrate that SUC2 and SUC1 display contrasting roles in roots.

## Materials and methods

### Plant material, growth conditions and harvests

Plants were grown in four interconnected Araponics^®^ boxes system (Araponics SA, Liège, Belgium). Nutrient medium was propelled from the nutrient growth container to the Araponics^®^ boxes by an electric pump (New-Jet 400, Aquarium Systems, Grabs, Switzerland). Nutrient medium was oxygenated with an air pump (Rena Air 100, Rena) and was replaced each week by fresh medium. Plants were cultivated in a phytotron, at 23 °C/18 °C, 10 h/14 h day/night (light 9 h to 19 h, dark, 19 h to 9 h) with 80 µmol m^−2^ s^−1^ light intensity, and 50%/70% day/night relative hygrometry. Arabidopsis seeds (*Arabidopsis thaliana* Col-0) were sown in Araponics^®^ seed holders in a drop of nutrient agar medium (0.65% agar, Musrashige and Skoog medium, M0233, Duchefa Biochemie) deposited on top of an inserted piece of autoclaved rockwool. Plants were harvested at six principal growth stages as defined in (Boyes et al. 2001): Young (Y; *D* + 31), Adult stage (A; *D* + 48), Inflorescence emergence (IE; *D* + 60); Siliques ripening (SR; *D* + 98) and Siliques harvest (SH; *D* + 125). For the diel experiment, only the young stage (Y) was studied. Root and rosette were harvested successively at 9, 13, 17, 21, 1 and 5 h, and frozen in liquid nitrogen for RNA and sugar extractions. For sampling during the night (1 and 5 h), all manipulations were conducted under non-actinic green light to avoid any light effect.

For plant development experiments, six harvests were performed, at the middle of the day, for the six stages of development studied (Y), (A), (IE), (F), (SR) and (SH). Root, rosette, stem and siliques were separately harvested and frozen in liquid nitrogen for RNA and sugar extractions. Biomass repartition and root shoot ratio (*R/S*) were calculated from dry weight (DW).

**Fig. 1:**
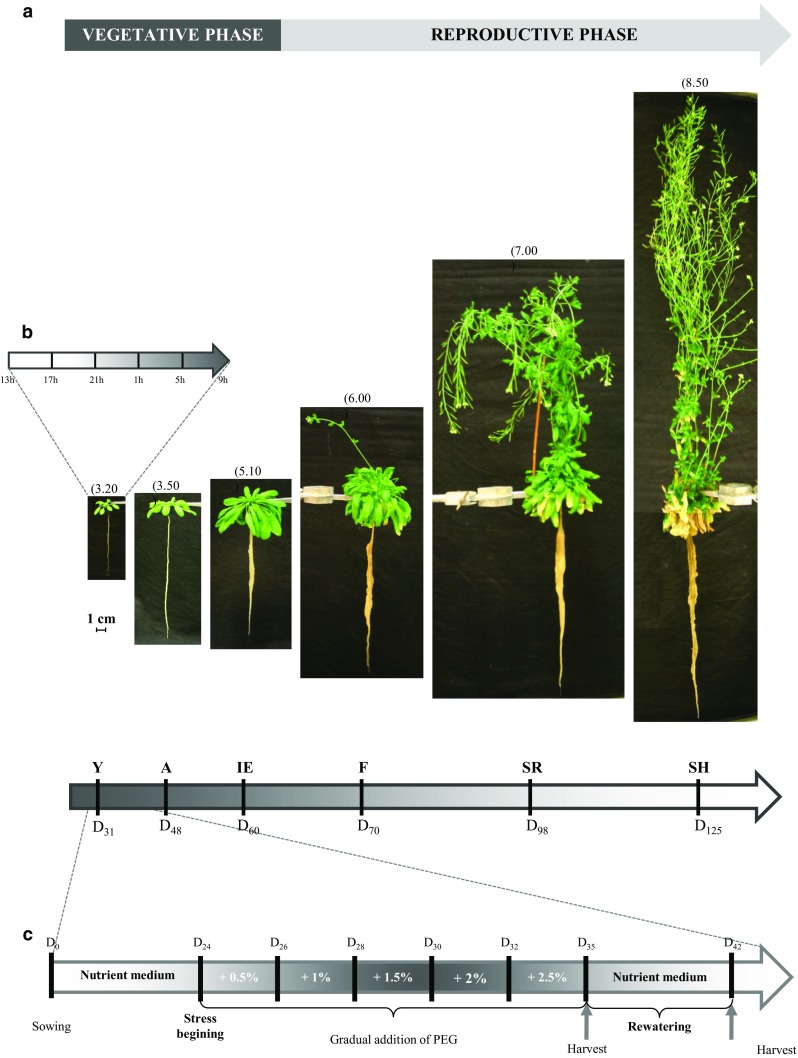
Design of the three experimental protocols used to study sucrose transporter genes expression and sugar allocation in source and sinks organs of *A. thaliana* grown hydroponically. Photographs of plants (*A. thaliana* ecotype ‘Columbia’) at the six principal growth stages in the hydroponic system: Y young stage, *D* + 31 after sowing; A adult stage, *D* + 48 after sowing; IE inflorescence stage, *D* + 60 after sowing; F flowering stage, *D* + 70 after sowing; SR silique ripening, *D* + 98 after sowing and SH silique harvest, *D* + 125 after sowing. The different stages are defined as described in Boyes et al. (2001) and indicated on the top of each photograph (**a**). Y, A, and IE correspond to the vegetative phase, and F, SR, and SH correspond to the reproductive phase of *A. thaliana* plant development. Plants were harvested at each time point to perform the ‘developmental experiment’. Schematic representation of the 24-h cycle protocol applied on 31 day-old plants (young stage, Y) (**b**). Roots and rosettes of plants were harvested successively at 9, 13, 17, 21, 1, and 5 h (light 9–19 h, dark, 19–9 h). Schematic representation of the osmotic stress protocol applied (**c**). After sowing, stressed plants grew in nutrient medium during 24 days. Osmotic stress was applied by gradual addition of polyethylene glycol (+ 0.5% PEG) in nutrient medium every 2 days, from *D* + 24 to *D* + 32, until the medium reached 2.5% PEG. During the rehydration phase starting at *D* + 35, medium with PEG was removed and control medium was added. At the end of the stress (*D* + 35) and after the rehydration phase (*D* + 42), control and stressed plants were harvested (*n* = 5) for further analyses

For osmotic stress experiments, stress was applied on 24-day-old plants, by gradual addition of 0.5% of polyethylene glycol (PEG) in nutrient medium every 2 days (Fig. 1c). The addition of PEG starts from *D* + 24 and was continued to *D* + 32 until the medium reached 2.5% PEG. This last concentration was maintained during 3 days, from *D* + 32 to *D* + 35. Then, the osmotic stress phase was followed by a rewatering phase, where medium with PEG was replaced by fresh control medium for 1 week, from *D* + 35 to *D* + 42. Harvests were performed, before stress application (*D* + 24), after osmotic stress phase (*D* + 35) and after rewatering phase (*D* + 42) (Fig. 1c). Roots and rosettes were separately harvested and frozen in liquid nitrogen for RNA and sugar extractions. Additional rosettes were also collected to measure physiological parameters (biomass, water status).

### Biomass repartition, root/shoot ratio (*R/S*), leaf area and growth rate

For the development experiments, plants from two independent experimentations have been used to determine dry weight, biomass repartition and root/shoot (*R/S*) ratio.

For the osmotic experiments, rosettes and roots DW was obtained (*D* + 24, *D* + 35 and *D* + 42) from three independent experimentations. The shoot growth rate (SGR) was then calculated after stress and rewatering (SGR = (DW_t2_ − DW_t1_)/(*t*2 − *t*1)). Root growth rate (RGR) was also calculated (RGR = (DW_t2_ − DW_t1_)/(*t*2 − *t*1)). *R/S* ratio was obtained by dividing roots average dry weight by rosette average dry weight.

The projected leaf area (PLA) was determined using pictures of plants with the threshold color plugin of ImageJ software (http://imagej.nih.gov/ij/). Leaf expansion rate (LER) was then calculated after stress and rewatering (LER = (PLA_t2_ − PLA_t1_)/(*t*2 − *t*1)).

### Water content (WC), osmotic potential

For the osmotic stress experiments, rosette water status was studied by performing water content (WC) and osmotic potential (*Ψ*_osm_) measurements.

Water content was determined as followed. Fresh weight (FW) was scored immediately after excision from the root. Dry weight (DW) was scored after 24 h at 80 °C. WC was determined as WC = (FW − DW)/FW.

*Ψ*_osm_ was measured on three to five excised leaves per plant, pooled in 2 mL syringes. The syringes were successively frozen in liquid nitrogen and thawed at room temperature three times. Sap was then extracted and collected in 15 mL tubes by centrifugation (8000*g*, 10 min). Ten microliters of the resulting sap was analyzed using a vapor pressure osmometer (Wescor Vapro 5520). *Ψ*_osm_ was calculated from osmolarity using the Van’t Hoff equation at 23 °C.

### RNA extraction and cDNA synthesis

Samples were ground in liquid nitrogen with a bead mill (TissueLyser II, Qiagen). Total RNA was extracted as described by (Kay et al. 1987). RNA quantity and quality were, respectively, checked by optical density at 260 nm and agar gel electrophoresis. cDNA was synthetized from 1 µg of total RNA after DNAse treatment (Sigma-Aldrich) using MML-V reverse transcriptase (Promega).

### Gene expression analysis

A set of 16 genes coding for sucrose transporters from two distinct families, the Sucrose Carriers (*AtSUC1, AtSUC2, AtSUC3, AtSUC4, AtSUC5, AtSUC6, AtSUC7, AtSUC8, AtSUC9*) and the Sugar Will Eventually Exported Transporters clade III (*AtSWEET9, AtSWEET10, AtSWEET11, AtSWEET12, AtSWEET13, AtSWEET14, AtSWEET15*) have been studied in roots, rosette, stem and siliques. An initial screen for these genes was performed by RT-PCR after 40 cycles using GoTaq Flexi DNA Polymerase (Promega) (95 °C, 30 s; 60 °C, 30 s; 72 °C, 30 s) and was analyzed on 2% agar gel electrophoresis. For further analyses by RT-qPCR, only genes that showed amplification in RT-PCR were studied. Quantitative PCR has been performed on 96 well-plate with a MasterCycler Realplex^2^ (Eppendorf) using GoTaq qPCR Master Mix (Promega). Relative expression has been determined according to the $2^{{ - \Delta {\text{C}}_{t} }}$ method. Relative expressions were compared to the adult stage for roots and rosette, to the flowering stage for stem and to the silique ripening stage for silique. Target gene expression was normalized to the expression of the plant gene *At5g12240* (Czechowski et al. 2005). The primers used are presented in Table S1.

### Soluble sugars and starch quantification

Glucose, fructose and sucrose were extracted from approximately 10 mg of tissue sample previously lyophilized, by three washings (1.5 mL and twice 0.5 mL) in methanol:chloroform:water (MCW) (12:5:3, by vol.). Supernatants containing soluble sugars were pooled and mixed with 0.6 volume of water and centrifuged (Landouar-Arsivaud et al. 2010). The upper aqueous phase was collected and evaporated at 30 °C with a MiVac Quattro (Genevac, Ipswich, UK). Soluble sugars were resuspended in 500 µL of water and quantified using sucrose/fructose/d-glucose Assay Kit (Megazyme). Starch content was measured from the pellet obtained after MCW washings using Total Starch HK Assay Kit (Megazyme).

### Transport of [U-^14^C] sucrose

Individual plants were transferred to 50 mL vials filled with nutrient medium the day before the experiment. Two microliters of 0.5 mM [U-^14^C] sucrose (18.5 kBq) (Perkin Elmer) dissolved in Mes buffer (10 mM, pH 5.5) were applied on a source leaf gently abraded with carborundum (∅ 60 µm, Prolabo). Transport of [U-^14^C] sucrose was performed for 5 h, with a 80 µmol m^−2^ s^−1^ light intensity. After 5 h of transport, rosette was immediately cut from root and stem. Radiolabelled source leaf was isolated from the rosette, and all the organs were placed between a 3MM Whatman^®^ paper and a mylar sheet, and then lyophilized for 1 week. Then, plants were exposed for 1 week on PhosphorImager screens (Storage Phosphor Screen, Molecular Dynamics), and then scanned with a 200 µm per pixel resolution (Scanner Typhoon, GE Healthcare).

To quantify radioactivity transported, each organ was incubated during 24 h in a digestion buffer (perchloric acid:hydrogen peroxide 30%:Triton X100 0,1%, 56:17:27, by vol.) at 55 °C. Scintillation cocktail (Ecolite) was then added and samples were counted for radioactivity (Tri-Carb2910, Perkin Elmer). Radioactivity was also counted in liquid culture medium.

## Results

### Hydroponic growth of *A. thaliana*

The aim of this work was to measure different physiological, biochemical and molecular parameters in *A. thaliana* roots to gain information on source-to-sink carbon balance in different growth conditions. For this purpose, the hydroponic growth system Araponics©, a convenient and versatile system, was used. The system is easy to handle and can produce a large amount of plants (Gibeaut et al. 1997) where the entire root system is readily accessible (Fig. 1a) (Siedlecka and Krupa 2002). Growth conditions were tightly controlled and different parameters of the culture medium were followed during the growth period of plants. The temperature in the nutrient growth container varied between 20 and 24 °C during the day and 18 °C during the night, which was inside the optimal temperature range for root growth (16–25 °C) described by Gibeaut et al. (1997). The nutrient medium was changed every week and the pH of the solution was followed. The pH of the fresh medium was 5.7 and increased between + 0.2 and + 0.7 pH unit after 7 days, depending on the age of plants. The alkalinisation of the rhizosphere is classically observed due to nitrate assimilation by roots (Stitt 1999). All the physical parameters measured here are in the range of values reported for hydroponically grown plants (Arteca and Arteca 2000; Siedlecka and Krupa 2002) and were very stable.

### An insight into diel regulation

#### Diel turnover of sugar content and expression pattern of a set of *AtSUC* and *AtSWEET* genes in *A. thaliana* during a 24 h cycle

**Fig. 2:**
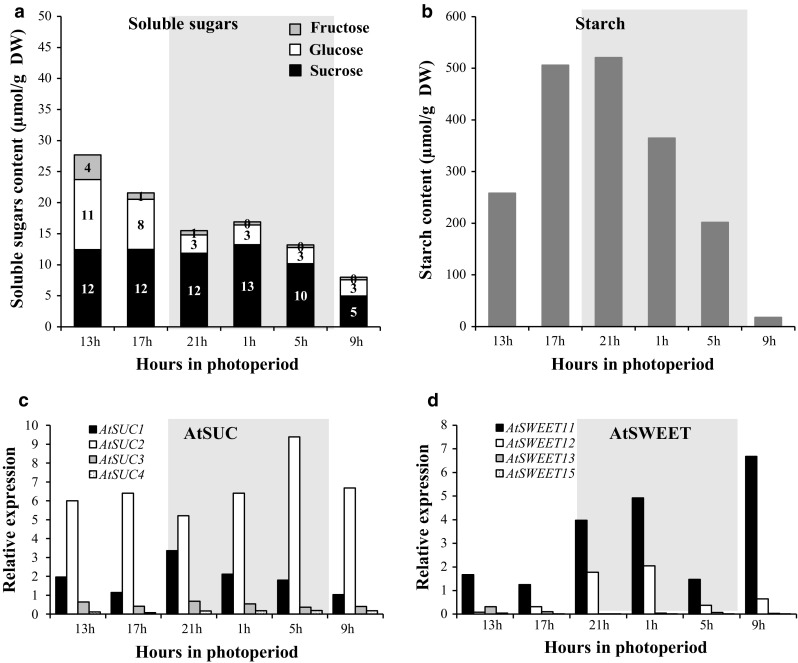
Sugar content and expression pattern of sucrose transporter *AtSUC* and *AtSWEET* genes in leaves during a 24-h cycle in 31-day-old *A. thaliana* plants grown hydroponically. Measurements of sucrose (black), glucose (white), fructose (light grey) (**a**), and starch contents in leaves (**b**). Data are expressed as the mean of measures obtained from pools of five plants. Relative expression of *AtSUC1*–*4* (**c**) and of *AtSWEET11, 12, 13,* and *15* (**d**) was determined by RT-qPCR. Data are expressed as normalized expression (no unit) to the reference gene *At5g12240* expression level (Czechowski et al. 2005) and are the mean of measures obtained from pools of five plants

**Fig. 3:**
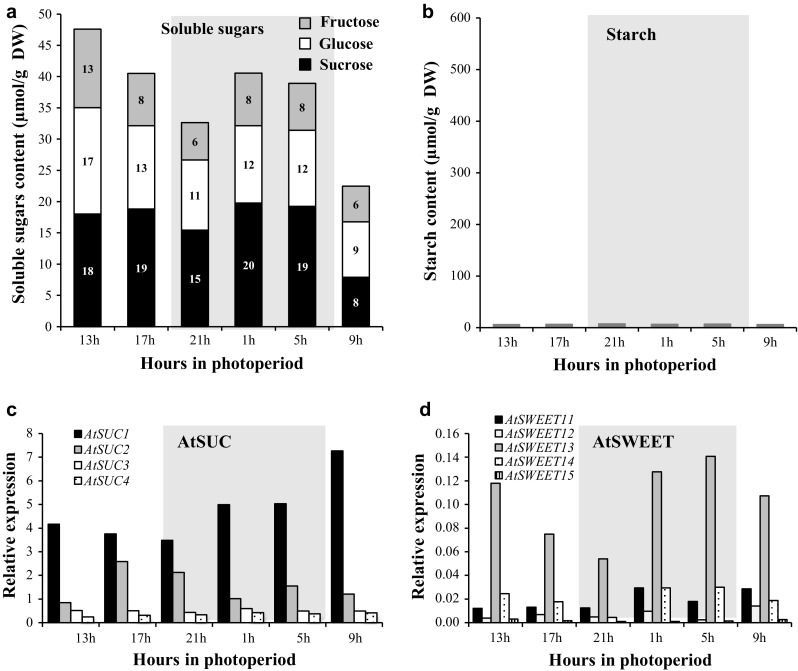
Sugar content and expression pattern of sucrose transporter *AtSUC* and *AtSWEET* genes in roots during a 24-h cycle in 31-day-old *A. thaliana* plants grown hydroponically. Measurements of sucrose (black), glucose  (white), fructose (light grey) (**a**), and starch contents in roots (**b**). Data are expressed as the mean of measures obtained from pools of five plants. Relative expression of *AtSUC1*–*4* **(c)** and *AtSWEET11*–*15* in roots (**d**) was determined by RT-qPCR. Data are expressed as normalized expression (no unit) to the reference gene *At5g12240* expression level (Czechowski et al. 2005) and are the mean of measures obtained from pools of five plants

To evaluate the light/dark variation of C balance in rosettes and roots, the concentration of the three major soluble sugars (sucrose, glucose and fructose) and starch was determined for each time point studied. Thirty-one-day-old plants were harvested every 4 h over a 24 h period (0900, 1300, 1700, 2100, 0100, and 0500 h) (Fig. 1b). Plants received light for 10 h (from 0900 to 1900 h). At this stage of development considered as young, rosette was composed of mature source leaves and young leaves (sink organs) and roots were the only true sink organs on plants. To identify the sucrose transporter genes expressed in rosette and roots, we examined the temporal pattern of *AtSUC* and *AtSWEET* genes expression in the same samples. The expression study of the nine *AtSUC* and seven *AtSWEET* genes (clade III members that have been reported to transport sucrose, Chen et al. 2012) identified in Arabidopsis was initially performed by PCR (Tables S1 and S2). To get more information about the regulation of their expression during a diel cycle, only genes detected by PCR were further analyzed by RT-qPCR (Figs. 2, 3).

#### Turnover in leaves

In leaves, the total soluble sugars content was higher at 1300, 4 h into the light cycle, during the peak of photosynthesis activity and decreased until the beginning of the dark period (2100 h) (Fig. 2a). After, the amount of sugar remained stable during the night until 0500 h and then, presented its lowest level at the end of the dark period (0900 h). Sucrose was the major soluble sugar in rosette and its amount remained unchanged between 1300 and 0500 h, but at the end of the dark period (0900 h), a twofold decrease (10–5 μmol/gDW) was noticed. The circadian variations of the total soluble sugars observed in leaves were due to hexoses, mainly glucose. Glucose strongly accumulated in the middle of the photoperiod (11 μmol/gDW at 1300 h), then decreased (1700 h) to reach its lowest level (3 μmol/gDW) at the beginning of the dark period (2100 h) and remained stable. Fructose level followed the same pattern but was constantly much lower than glucose content. Concerning starch, its amount rose in leaves during the day to reach its maximum level (520 μmol/gDW) early in the dark period (2100 h). Then, starch was continually degraded to reach a very low amount (17 μmol/gDW) at the end of the dark period, indicating that starch turnover was almost complete at this point (Fig. 2b).

Among the nine *AtSUC* genes studied, four: *AtSUC1*, *AtSUC2*, *AtSUC3* and *AtSUC4* were found to be expressed in rosette (Fig. 2c, Table. S2). *AtSUC1* and *AtSUC2* genes were detected at a higher expression level than *AtSUC3* and *AtSUC4* (Fig. 2c), which stayed similar and low all along the diel cycle. Actually, *AtSUC2* was the most expressed *AtSUC* gene in the rosette, displaying a high and stable diurnal expression. After a slight decrease at the beginning of the dark period (2100 h), its expression rose again during the night (21–5 h) to reach its maximum level at 0500 h. Regarding *AtSUC1* gene, its expression was lower than that of *AtSUC2* and, its transcripts level remained stable during the circadian cycle, with a slight increase at the beginning of the dark period from 2100 to 0100 h (Fig. 2c).

Concerning the *AtSWEET* genes, four of them were expressed in rosettes: *AtSWEET11, AtSWEET12, AtSWEET13* and *AtSWEET15* (Fig. 2d, Table. S2). During the diel cycle, a very low expression level of both *AtSWEET13* and *AtSWEET15* was noticed (Fig. 2d) and even if the expression of *AtSWEET11* was higher than that of *AtSWEET12*, both genes presented a highly similar diel cycle pattern of expression. Their level of expression remained stable during the light period, then increased during the first half of the dark period (2100 and 0100 h) followed by a reduction during the second half of the dark period but surprisingly, a high increase of *AtSWEET11* expression was noticed at 0900 h, its maximal expression level (Fig. 2d).

#### Turnover in roots

In roots, total sugar content (on a DW basis) was consistently higher than in leaves but decreased from 1300 to 2100 h, as observed in leaves. This fall was linked to a decrease of glucose (17 μmol/gDW to 11 μmol/gDW) and fructose (13–6 μmol/gDW) levels in roots, while sucrose remained stable. Indeed, the sucrose level was stable from 1300 to 0500 h and decreased at the end of the dark period (0900 h), as already noticed in leaves. During the dark period, the total sugar content increased first, then decreased to reach the lowest level measured over the diel cycle (Fig. 3a). In contrast to leaves, the part of each soluble sugar in total amount was constant in roots at every harvest time points. Furthermore, the amount of starch in roots was very low (around 6–7 μmol/gDW), compared to leaves and did not show noticeable variation during the circadian cycle (Fig. 3b).

In roots, the same four *AtSUC* genes as in leaves were identified as being expressed (Figs. 2c, 3c). The expression pattern of both *AtSUC3* and *AtSUC4* genes remained stable and low, while *AtSUC1* and *AtSUC2* were the most highly expressed *AtSUC* genes (Fig. 3c), *AtSUC1* being more highly expressed than *AtSUC2* in the roots, contrary to their expression levels in leaves. Its expression was high during the light period and increased during the dark period to reach a maximum level at the end of the dark period (0900 h).

The same four *AtSWEET* genes were expressed in rosettes and roots: *AtSWEET11, AtSWEET12, AtSWEET13* and *AtSWEET15*, while *AtSWEET14* transcripts were specifically present in roots (Fig. 3d, Table. S2). All the *AtSWEET* genes detected in roots (*AtSWEET11*-*15*) were less expressed than in shoots but *AtSWEET13* was constantly the most expressed gene (Fig. 3d).

### Source-to-sink C partitioning during *A. thaliana* development

#### Plant growth during a full life cycle in hydroponic system

The hydroponic culture system used in this experimentation allowed the full life cycle of *A. thaliana* (Col-0) plants from germination to seed harvest. In this experiment, six main stages of development were studied after sowing (*D* = 0): young stage (Y; *D* + 31), adult stage (A; *D* + 48), inflorescence emergence stage (IE; *D* + 60), flowering stage (F; *D* + 70), silique ripening (SR; *D* + 98) and silique harvest (SH; *D* + 125) (Boyes et al. 2001). The three first stages corresponded to the vegetative phase, while the others represented the reproductive phases (Fig. 1a). The transition between both phases appeared at the inflorescence stage (*D* + 60). Plants at the young stage (Y) were the same as those tested for the circadian experiment.

For each stage of development, the growth of rosette (photosynthetic source organ), root (sink organ) and stem (if present, sink organ with flowers and seeds) was estimated by the measurement of organ dry weight (DW) (Table S3). Roots and rosette DW increased up to *D* + 70 (F stage). Then root DW increased more slowly and rosette DW tended to level off. Stem DW increased dramatically to become the biggest organ at seed harvest, *D* + 115 (Table S3). The shoot (rosette plus inflorescence) to the root ratio increased from the Y to the SH stages of development with a greatest rise up at *D* + 70, corresponding to the inflorescence growth on stem (Table S3).

**Fig. 4:**
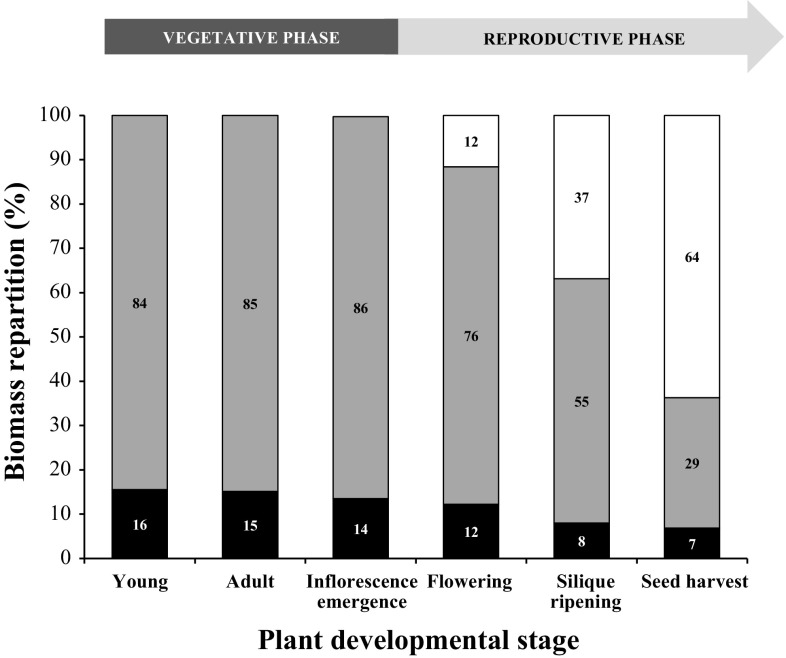
Biomass partitioning in the different organs of plants at the six principal growth stages of *A. thaliana* grown hydroponically. Three organs have been considered: roots (black), rosette (grey), and stem (white, when present). The percentage of biomass has been calculated from the mean of dry weight of seven to eight individual plants from two independent experiments. A biomass of 100% corresponds to the sum of the dry weight of organs present on the plants at the developmental stage considered

The relative proportion of each organ was calculated from their DW (Fig. 4). Up to the (IE) stage, the biomass distribution between rosette (84–86%) and roots (16–14%) was stable. At (F) stage, when inflorescences grew, the rosette contribution decreased to 76%, while the root contribution remained close to former values (12%). At this stage, roots and stems each accounted for 12% of the total plant weight. For the two last stages (SR, SH), the contribution of the stem to the total weight increased to become the biggest organ of the plant (64% of the plant DW). Conversely, the rosette biomass contributed less and less to the total weight of the plant. Concerning roots, their proportion declined at the (SR) stage (8%), but remained stable at the (SH) stage (Fig. 4).

#### Sugar partitioning and evolution of the expression pattern of a set of specific *AtSUC* and *AtSWEET* genes in rosette, roots, stem and siliques during a full *A. thaliana* life cycle

**Fig. 5:**
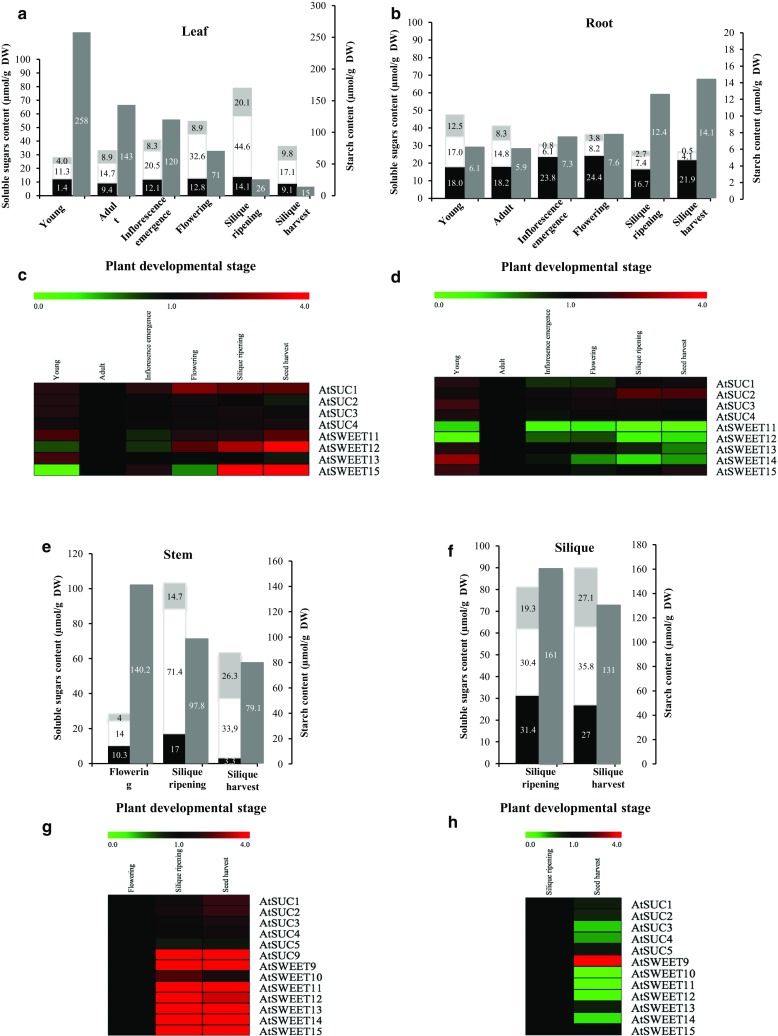
Sugar content and fold changes of expression for a set of *AtSUC* and *AtSWEET* genes in different organs and at the six principal growth stages of *A. thaliana* grown hydroponically. Sucrose (black), glucose (white), fructose (light grey), and starch (dark grey) contents measured in leaves (**a**), roots (**b**), stems (**e**), and siliques (**f**). Data are expressed as the mean ± SE of measures obtained from five plants. Fold change of expressed *AtSUC* and *AtSWEET* genes in rosettes **(c**), roots (**d**), stems (**g**), and siliques (**h**) has been studied. Fold change values are displayed as a two colours heat-map view (MeVsoftware (http://www.tm4.org/mev.html), with rows corresponding to the genes of interest and columns to the six development stages studied with). The oligonucleotides primers used in RT-qPCR experiments are presented in Table S1. Fold change values are obtained by comparison with the adult stage after normalization to the reference gene *At5g12240* (Czechowski et al. 2005). Data are the mean of three measures, each corresponding to a pool of five plants

The amount of the three major soluble sugars and starch was determined in rosette, roots, stems and siliques at the six stages previously described. Plants were collected at 1300 h, when total sugar amount was highest in source leaves (Fig. 5a). After determining diel regulated expression of the main sucrose transporters (*SUCs* and *SWEETs*) genes in rosettes and roots, the same approach (RT-PCR, 40 cycles) was followed to identify genes expressed in the rosette and roots and in two news organs: stem and siliques (Table S1). The regulation of genes by RT-qPCR was conducted only on genes detected by PCR (Table S2). Fold change values (Table S2) were obtained by comparison with the adult stage (A) for rosette and roots (Fig. 5c, d), with the flowering stage (F) for stems (Fig. 5g) and finally, ripening stage (SR) for siliques (Fig. 5h.) In parallel to the sucrose transporter gene expression, the expression of two main genes implicated in photosynthesis (*AtRBCS* and *AtCAB1*) and one gene involved in leaf senescence *(AtSAG12*) were also followed to estimate the physiologic status of leaves during our experiment (Fig. S1).

#### Sugar partitioning in leaves

In rosettes, total soluble sugars increased from the (Y) stage to the (SR) stage then decreased dramatically at the last stage (Fig. 5a). Glucose accumulated gradually between the (Y) and (SR) stages, with a larger increase at the (F) stage corresponding to the appearance of a new sink organ (stem). At the last stage (SH) where seeds were fully filled, glucose content decreased considerably. Actually, glucose was the major soluble sugar in leaves, except at the (Y) stage. Fructose also accumulated in rosette but to a lesser extent and decreased at the last stage. Sucrose level presented less variation in leaves during plant development (Fig. 5a). The highest level of starch was found at the (Y) stage and decreased continually during plant development especially from the (F) stage (Fig. 5a).

Among the four *AtSUC* genes (*AtSUC1*–*4*) expressed in rosettes, only *AtSUC1* displayed a higher expression at the flowering stage (F). Both *AtSUC2* and *AtSUC3* appeared slightly more expressed at the young stage (Y) than during the five other stages with a steady strong level of expression of *AtSUC2*. Besides, *AtSUC4* displayed the same expression level at all stages (Fig. 5c and Table S2). Concerning *AtSWEET* genes in the rosette (*AtSWEET11*–*13* and *AtSWEET15*), three of them, *AtSWEET11, AtSWEET12* and *AtSWEET15* presented a similar level of expression with an increase at the two last stages of development, *AtSWEET13* showing the same level of expression at the six stages of development (Fig. 5c and Table S2). The measurement of the *AtRBCS* and *AtCAB1* expression in leaves indicated that the level of both transcripts decreased from the (IE) stage until the end of the plant development For the senescence marker gene *AtSAG12*, its expression presented a strong increase at the two last stages of development (SR and SH) (Fig. S1 and Table S4).

#### Sugar partitioning in roots

In roots, sucrose was the major sugar and its level remained high and stable in this sink organ, with a slight increase at the reproductive phase. Fructose and glucose showed their highest levels during the two first stages of development studied (Y and A) but decreased after (IE) and remained stable (Fig. 5b). It is interesting to notice that from the (IE) stage, the total soluble sugar amount (on a DW basis) in roots became lower than in leaves, the amount of starch in roots was weak and stable until the (F) stage and then almost doubled at the two last stages (Fig. 5b). However, as already mentioned, the content of starch in roots was smaller than in shoots, whatever the stage considered. Among the four *AtSUC* genes (*AtSUC1*–*4*) expressed in roots, only *AtSUC2* presented a noticeable increased expression during the two last stages (SR and SH). Concerning *AtSUC1*, the most expressed sucrose transporter gene in roots, its expression was high and stable with only a slight decrease at the (IE) and (F) stages. (Figure 5d and Table S2). All the *AtSWEET* genes identified in roots (*AtSWEET11*–*15*) were found expressed at the four first development stages (Y, A, IE and F), while at the (SR) and (SH) stages, the expression of the *AtSWEET11* gene was not detected anymore. All those five *AtSWEET* genes showed a weak transcript level, except during the (Y) stage, when *AtSWEET13*–*15* were detected at a higher level than at the (A) stage (Fig. 10 and Table S2). As observed in the circadian experiment at the (Y) stage, *AtSWEET13* remained the most highly expressed *AtSWEET* gene in roots (Fig. 5d and Table S2).

#### Sugar partitioning in stems

In stems, sugars were measured from the (F) stage to the (SR) stage. At the (F) stage, the stem was very short (Fig. 1a) and can be considered as a full sink organ. However, the soluble sugars content was low and closed to the one observed in leaves at the vegetative stage [Y, A and IE; (Figs. 5a, e)]. Conversely at the (SR) stage, the sugar level increased dramatically, especially due to glucose content, and displayed the highest amount found in the four organs studied during plant development (Fig. 5). At a lesser extent, fructose level also increased. At this stage corresponding to the filling of the seeds, the stem could be considered as a dominant source organ as its sugar pattern was closed to the one observed at SR stage in rosettes (Fig. 5e). At the last stage studied (SH), where the seeds are fully filled, the sucrose level dramatically decreased in the stem and the glucose amount was half of that observed at the (SR) stage as observed in rosettes. It is noticeable that starch content slightly decreased in stem until the (SH) stage, but remained high (Fig. 5e).

Concerning sucrose transporter gene expression in stems, six *AtSUC* (*AtSUC1*–*5* and *AtSUC9*) genes were found expressed. *AtSUC1* presented a relatively high level of expression and *AtSUC2* expression increased at the three last stages of development (F, SR and SH). *AtSUC9* expression started in stem at the two last stages (SR and SH) and remained low, as observed for *AtSUC3*–*5* (Fig. 5g and Table S2). In stems, among the seven *AtSWEET* genes expressed, *AtSWEET11*–*15* presented an increasing level of expression at the three stages studied. *AtSWEET11*–*13* were the most expressed genes while transcripts level of *AtSWEET10* decreased at (SH) and *AtSWEET9* started to be expressed at (SR) stage (Fig. 5g and Table S2).

#### Sugar partitioning in siliques

In siliques containing seeds, considered as a true sink organ like roots, the soluble sugar content was high at both stages studied (SR and SH), and the partitioning of the three sugars was roughly constant as observed in roots at the Y and A stages (Fig. 5f). Concerning starch, its content was very high in siliques at both (SR) and (SH) stages (Fig. 5f).

In siliques, five *AtSUC* genes *(AtSUC1*–*5*, Fig. 5h) were expressed and at the (SR) stage (Table S2), *AtSUC2* displayed the higher level of expression among all the organs at all stages. Our results indicated also a high level of expression for *AtSUC1*, *AtSUC3* and *AtSUC5* at (SR), while their expression dropped at (SH) (Fig. 5h and Table S2). Concerning *AtSWEET* genes, six of them (*AtSWEET10*–*15*) presented a higher level of expression at (SR) compared to (SH), with a stronger level for *AtSWEET11*–*13* and *AtSWEET15* (Fig. 5h), *AtSWEET9* being only expressed at the last stage (Fig. 5h and Table S2).

#### Study of [U-^14^C] sucrose transport during *A. thaliana* development

To find out more about C allocation among the different organs, the distribution of [U-^14^C] sucrose fed to one source leaf was studied. The pattern of ^14^C allocation was studied after 5 h of transport, at each developmental stage. Preliminary experiments were run with different transport time (from 30 min to 5 h) and it was demonstrated that 5 h was sufficient to get consistent label of the whole root system (data not shown). The data (in %) represent the export of [U-^14^C] sucrose from one mature leaf (source) to the rosette (sink leaves), roots, stems (if present) and external medium bathing the roots.

**Fig. 6:**
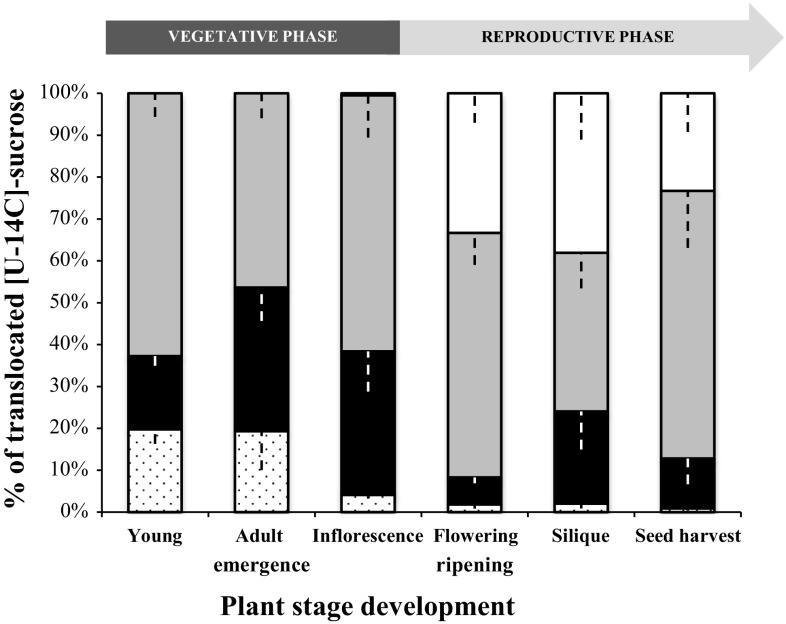
Quantitative representation of [U-^14^C] sucrose transport (expressed as % of total radioactivity exported) from a mature leaf to sink leaves, roots, stems (if present), and external medium and at the six principal growth stages of *A. thaliana* grown hydroponically. For each development stage studied, plants were fed with a drop (10 µl) of ^14^C sucrose on a mature leaf, after gentle scrubbing with carborundum. After 5 h of transport, the radioactivity was counted in the rosette (grey), stem (white), roots (black), and external medium (dots), and the distribution of radioactivity calculated among the three compartments. Each result is the mean ± SE of measures obtained from three to eight individual plants from two independent experiments

At the (Y) stage, ^14^C was measured in rosettes (65.5%), roots (17.5%) and in the external medium (19.8%) (Fig. 6). ^14^C found in the external medium was considered as carbon that has passed through the roots and excreted into the medium. Therefore, the total C translocated to the root was the sum of the radioactivity found in roots and external medium: for the (Y) stage, this value was 37.3%. At the (A) stage, the ^14^C amount decreased in rosettes (46.4%) for the benefit of roots (34.3%) and remained stable in the external medium (19.3%). ^14^C translocated to roots corresponded to 53.6% of the total, meaning that at this stage the C allocation was equal between rosette and roots, indicating that the root system was a major sink before flowering. At the (IE) stage, ^14^C partitioning between rosettes and roots was similar to the (Y) stage but a strong decrease in ^14^C was noticed in the external medium (4.1% of total).

During the reproductive phase, the amount of ^14^C in roots decreased drastically at the last three stages of development studied (F, SR and SH), (respectively, 6.5, 13.7, and 11.9%), as in the external medium (respectively, 1.8, 2.3, and 0.9%). These results indicated that less labelled C was exported from the source leaves to the roots. At the reproductive phase, a new sink organ has appeared: the stem, and C translocation was preferentially routed to the stem than to the roots. From the (F) stage, the amount of ^14^C measured in the stem was important (33.4%) and increased (38.8%) at the (SR) stage, highlighting C translocation from the source leaf to flowers and maturing fruits present on stems. Conversely, in the rosette, ^14^C was high (58.4%) at the (F) stage and decreased (37.8%) at the (SR) stage, but ^14^C was less exported to the stem (23.3%) at the last stage (SH), and preferentially allocated to the rosette (63.9%) (Fig. 6).

### Impact of an osmotic stress on the source/sink allocation of C

#### Impact of osmotic stress and rewatering on the phenotype and water status of source/sink organs

As water deficit is known to alter plant physiology and carbohydrates redistribution between source and sink organs (Hummel et al. 2010; Durand et al. 2016) the impact of osmotic stress was studied on hydroponically grown *A. thaliana*. The study was performed on 24–42 day-old plants while one source (rosette) and one sink (root) were present (Fig. 1a). To mimic water stress in hydroponically grown plants, an osmotic stress was applied by gradual addition of PEG 6000 in the growth medium, starting 24 days after sowing (Fig. 1c). After 11 days of osmotic stress (2.5% final PEG), growth medium was substituted by medium without PEG, allowing a rewatering phase for the next 7 days. Plants were sampled at the end of the osmotic stress (*D* + 35) and rewatering periods (*D* + 42). The impact of the osmotic stress was estimated by analyzing some physiological and growth parameters.

The effect of osmotic stress was visible on rosette as the projected leaf area was smaller in stressed plants (26.8 cm^2^) than in control plants (46.3 cm^2^) (Table 1). This result was supported by the determination of the leaf expansion rate at the end of the stress period, which was reduced 2.2 fold in stressed plants (1.4 cm^2^ days^−1^) compared to control plants (3.1 cm^2^ days^−1^). After 1 week of rewatering, leaf expansion rate of stressed plants increased 1.8 fold compared to stressed plants harvested before rewatering. This highlighted that rosette growth resumed during rewatering, as projected leaf area increased from 26.8 cm^2^ to 44.4 cm^2^. Rosette dry weight was reduced after 10 days of osmotic stress (106 mg) compared to control plant (132 mg), corresponding to a leaf biomass reduction of 19.7%. Root weight was also reduced after osmotic stress (1.7 mg) compared to control roots (2 mg), i.e. a root biomass reduction of 15%. The shoot-to-root ratio was slightly lower in stressed plants (4.6) compared to the control plants (5.1), indicating that rosette growth was slightly more impacted by osmotic stress than root growth. After rewatering, the rosette biomass of stressed plants increased about 65% compared to plants at the end of stress. This result together with the increased leaf expansion rate, illustrated a physiological recovery of stressed rosettes. Surprisingly, for stressed roots, after 1 week of rewatering, the growth rate was still decreasing (− 11.8%) compared to plants at the end of stress (− 15%). As a result, the shoot-to-root ratio of stressed plants increased after 1 week of rewatering (5.3) compared to plants at the end of stress (4.6) to reach values similar to those of control plants (5.1 for C_st_; 5.6 for C_rew_) (Table 1).

**Table 1:** Impact of osmotic stress and rewatering on a set of physiological parameters

Parameters	Unit	Treatment			
		Osmotic stress phase		Rewatering	
		Control (Ctrl)	Stressed (St)	Control (Ctrl)	Stressed (St)
Projected leaf area	cm^2^	46.3 (11.6)	26.8 (7.8)	81.1 (20.1)	44.4 (17.1)
Leaf expansion rate	cm^2^ days^−1^	3.1	1.4	5	2.5
Rosette dry weight	mg	132 (32)	106 (34)	302 (88)	175 (52)
Rosette growth rate	mg days^−1^	10.3	7.9	24.4	9.9
Root dry weight	mg	25.6 (1.6)	23.1 (7.0)	53.8 (8.9)	33.2 (14.0)
Root growth rate	mg days^−1^	2	1.7	4	1.5
Shoot–root ratio DW	mg shoot mg^−1^ root	5.1	4.6	5.6	5.3
Water content	g water g^−1^ fresh weight	0.92 (0.01)	0.89 (0.02)	0.92 (0.01)	0.91 (0.01)
Leaf osmotic potential	Mpa	− 0.88 (0.06)	− 1.33 (0.48)	− 0.9 (0.11)	− 0.92 (0.12)

The measurements (except growth rates) are performed 11 days after the onset of osmotic stress (*D* + 35) and 7 days after the beginning of rewatering (*D* + 42). Growth rate, leaf growth rate, and root growth rate are calculated during the osmotic stress phase (*D* + 24 to *D* + 35) and the rewatering phase (*D* + 35 to *D* + 42). DW is obtained from 31 (day 24 after sowing) and 26 plants (day 35 and 42 after sowing) per condition [control (Ctrl) and stressed (St)] from three independent experiments. Root dry weight is obtained from 11 (day 24 after sowing), 16 (day 35 after sowing) and 20 plants (day 42 after sowing) per condition (Ctrl and St) from three independent experiments. The projected leaf area is determined using picture of plants from three experiments, with the threshold color plugin of ImageJ software. Around 120 plants per condition [control (Ctrl) and stressed (St)] are used for day 24 and day 35 after sowing, and 70 for day 42 after sowing. Water content is determined from 15 whole rosettes per condition from three independent experiments. Osmotic potential is measured on three-to-five excised leaves per plant on 15 plants from three independent experiments. Three measurements are performed for each sample

To estimate the water status of plants, water content (WC) and osmotic potential were determined in rosettes. During osmotic stress, WC declined in stressed rosettes (0.89) compared to control ones (0.92), but returned to control level after one week of rewatering (0.91). Rosette osmotic potential was also lower in stressed plants (*Ψ*_leaf_ = − 1.33 MPa) than in control plants (*Ψ*_leaf_ = − 0.88 MPa). After rewatering, the osmotic potential in stressed plants was similar to the one of control plants (− 0.92 and − 0.90 MPa, respectively). These results (WC and *Ψ*_leaf_) indicated the complete recovery of hydration state in stressed plants after 1 week of rewatering (Table 1).

#### Impact of osmotic stress and rewatering on: sugar partitioning and the expression pattern of specific *AtSUC* and *AtSWEET* genes in rosettes and roots

**Fig. 7:**
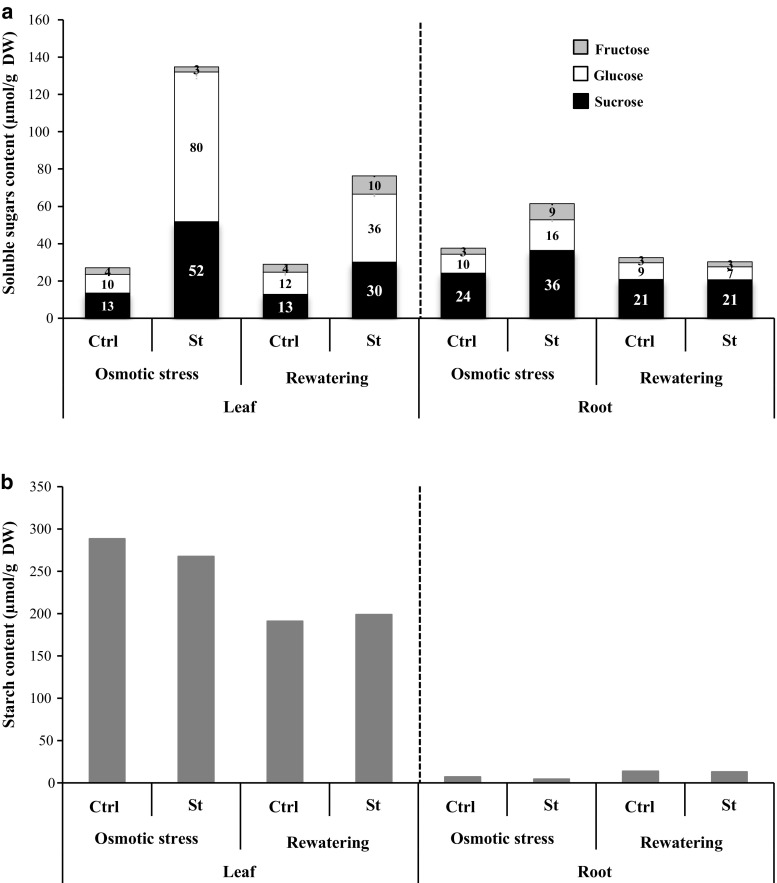
Impact of osmotic stress and rewatering on sugar and starch contents of rosette and roots of plants grown hydroponically. Sucrose (black), glucose (white), and fructose (light grey) contents (**a**) and starch content (**b**) measured in leaves and roots after osmotic stress and rehydration phase. Data are expressed as the mean of measures obtained from pools of plants

The analysis of soluble sugars, starch contents and sucrose transporters expression was performed in rosette and roots. In rosettes, a marked increase (around fourfold) of total soluble sugars content was observed at the end of the osmotic stress (Fig. 7). This increase was mainly due to sucrose and glucose levels (increased fourfold and sevenfold, respectively). After rewatering, soluble sugar content decreased in stressed rosettes, but remained still higher than in control rosettes (glucose, threefold; sucrose, 2.3-fold; fructose, 2.5 fold) (Fig. 7a).

In roots, sugar contents also increased after the stress period, but to a lesser extent than in rosettes. This increase was due to a higher level of the three soluble sugars. Indeed sucrose, glucose and fructose contents rose, respectively, about 1.5, 1.6 and 3 times. After rewatering, the amount of the three sugars recovered its control level (Fig. 7a).

Concerning starch its amount did not seem impacted by the osmotic stress, as its content was comparable in stressed (267 μmol/gDW) and control (288 μmol/gDW) rosettes (Fig. 7b). During the rewatering phase, even though starch content decreased in both stressed and control rosettes, starch content value was similar in stressed (198 μmol/gDW) and control rosettes (191 μmol/gDW) (Fig. 7b). In roots, starch content remained very low in all conditions.

**Fig. 8:**
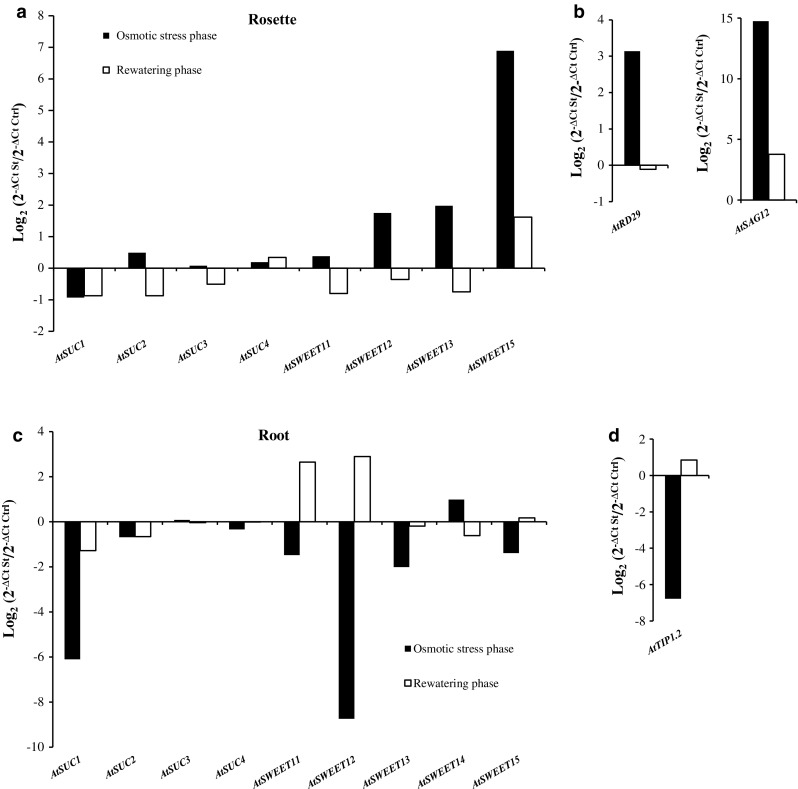
Impact of osmotic stress and rewatering on the relative expression (log_2_ St/C) of a selected set of *AtSUC* and *AtSWEET* genes in leaves and roots of plants grown hydroponically. *SUC* and *SWEET* transcripts level are quantified by RT-qPCR in rosette (**a**) and in roots (**c**) after osmotic stress (black) or rewatering (white). Values in each graph represent the log2 relative expression St/C measured by RT-qPCR using *At5g12240* (Czechowski et al. 2005) gene as reference and represent the mean of measures obtained from a pool of five plants The expression of specific genes as osmotic stress markers are followed in the leaf: *AtRD29* (**b**) and in the root: *AtTIP1.2* (**d**) or as senescence-associated gene: *AtSAG12* (**b**) during osmotic stress (black) and during rewatering phase (white)

The results of RT-qPCR experiments compared expression in stressed plants (St) with those in control plants (Ctrl) and were expressed as the Log2 relative expression $(\Delta {\text{C}}_{t}^{\text{St}} /\Delta {\text{C}}_{t}^{\text{Ctrl}} )$. After 10 days of osmotic stress, the expression of *AtSUC2*, *AtSUC3*, *AtSUC4* and *AtSWEET11* was not modified in rosettes (log2 < 1), while *AtSUC1* was slightly repressed (Fig. 8a). In contrast, three sucrose facilitators, *AtSWEET12*, *AtSWEET13* and *AtSWEET15* displayed a higher expression level in stressed compared to control rosettes and exhibited a strong expression after rewatering (Fig. 8a). The expression of the *AtRD29*, a specific osmotic stress marker gene (Xiong et al. 2006) increased after the stress and then returned close to the level of control rosettes after the period of rewatering (Fig. 8b). The expression of *AtSAG12*, a senescence marker gene (Gan and Amasino 1995) increased during the osmotic phase and decreased—but remained high—after rewatering (Fig. 8b).

In roots, *AtSUC3* and *AtSUC4,* genes showed the same level of expression after the stress and rewatering phases. The expression of *AtSUC1, AtSUC2, AtSWEET11, AtSWEET12, AtSWEET13* and *AtSWEET15* were repressed (Fig. 8c). *AtSUC1, AtSWEET13* and *AtSWEET15*, displayed a higher level of transcripts after the rewatering phase. Surprisingly, *AtSWEET11* and *AtSWEET12* expression appeared to be induced after rewatering (Fig. 8c). The osmotic stress was estimated in roots by following the expression of *AtTIP1.2* gene. *AtTIP1.2* is known as a specific osmotic stress repressed-marker in roots (Alexandersson et al. 2005) and was found repressed in stressed roots. After rewatering, the transcript level of *AtTIP1.2* was slightly induced (Fig. 8d).

#### Study of [U-^14^C] sucrose transport during osmotic stress and rewatering in rosettes and roots of *A. thaliana*

**Fig. 9:**
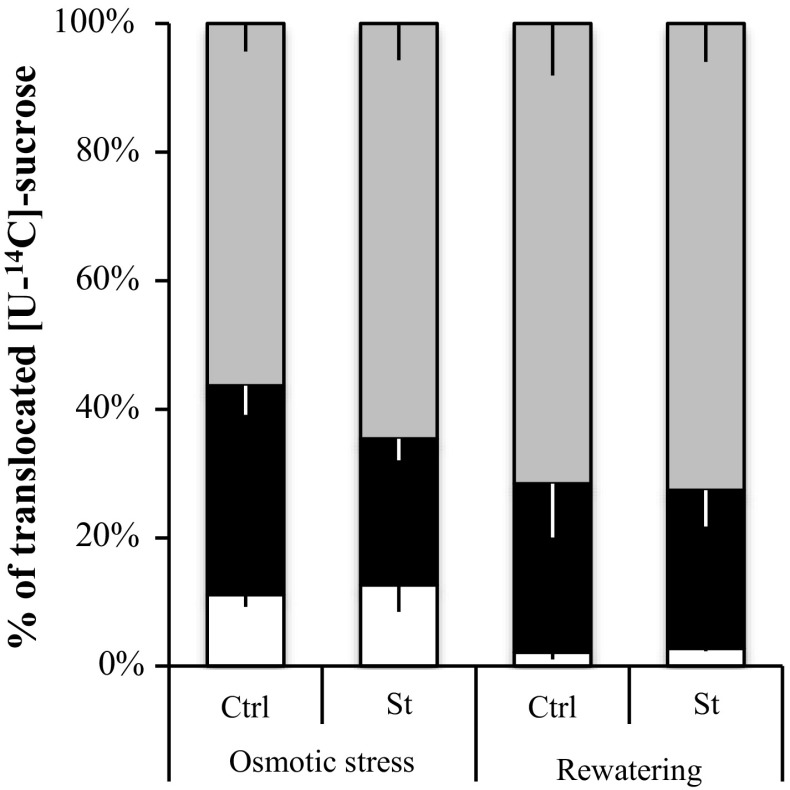
Quantitative representation of [U-^14^C] sucrose transport (expressed as % of total radioactivity exported) from a mature leaf to sink leaves, roots, and external medium. For each condition studied (osmotic and rewatering phases), plants are fed with a drop (10 µl) of ^14^C sucrose on a mature leaf, after gentle scrubbing with carborundum. After 5 h of transport, the radioactivity is counted in the rosette (grey), roots (black), and external medium (white) and the distribution of radioactivity calculated between the three compartments. Each result is the mean ± SE of measures obtained from three to eight individual plants from two independent experiments

C allocation in rosettes and roots was evaluated during osmotic stress followed by a rewatering phase of 1 week. The export of [U-^14^C] sucrose (% in Fig. 9 and DPM, Fig. S2) from one mature leaf to the rosette (sink leaves), roots and external medium bathing the roots was followed. Less ^14^C was recovered in stressed plants (St: 19037 DPM) compared to the control plants (Ctrl: 33109 DPM) (Fig. S2). The decrease of C transported in stressed plants reflected a lower level of C exported to both organs rosette and roots (Fig. S2). Even though less ^14^C was translocated into stressed plants, the sucrose partitioning between rosettes and roots and external medium remained stable in stressed and control plants (Fig. 9). After one week of rewatering, St plants displayed a higher amount of ^14^C transported (53500 DPM) compared to Ctrl plants (29713 DPM) (Fig. S2). The ^14^C partitioning in the different compartments studied indicated that, ^14^C ratio transported in rosette, roots and external medium in St (28, 25, and 73%, respectively) and Ctrl plants (22, 26 and 72%) was similar after a rewatering phase (Fig. 9).

## Discussion

### The hydroponic system culture: a useful tool to follow rosettes, roots, stems and siliques development and the expression of sucrose transporter genes

The hydroponic growth of plants allows a full development of *Arabidopsis* until seeds production (Fig. 1a). As shown in many others studies, hydroponically grown plants show the same morphological and physiological traits (Fig. 1a) as plants cultivated in soil (Gibeaut et al. 1997; Norén et al. 2004), as e.g. demonstrated for the inflorescences development during the reproductive phase (Arteca and Arteca 2000). Moreover, the main advantage of hydroponic culture allows harvest of undamaged clean root in large quantity (Arteca and Arteca 2000; Norén et al. 2004), and the collection of roots exudates for studying different kinds of environmental stress such as water deficit mimicked by a PEG osmotic stress (Arteca and Arteca 2000; Yang et al. 2013).

In this study we first intended to better understand the implication of sucrose transporter genes in source–sink relationship, during full *Arabidopsis* life cycle. Using this culture system, we performed a comprehensive expression profiling of sucrose transporters genes (nine *AtSUC* and seven *AtSWEET* genes of the clade III members) in four main organs at six defined developmental stages of *A. thaliana* plants (Fig. 1a). In addition, we studied the regulation of their expression during a 24 h cycle (on plants corresponding of the Y stage) (Fig. 1b) and during an osmotic stress (on 35-day-old plants) in both source (leaves) and sink (roots) organs (Fig. 1c).

### Expression of *SUC* and *SWEET* transporter genes in rosettes, roots, stems and siliques of *A. thaliana*

**Fig. 10:**
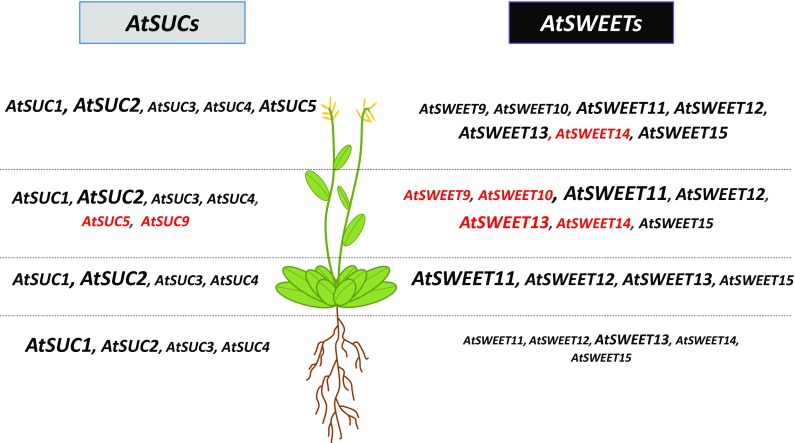
Expression pattern of a selected set of *AtSUC* and *AtSWEET* genes in different organs of plants and at the six growth stages of *A. thaliana* grown hydroponically. Representation of preferential *AtSUC* and *AtSWEET* genes expression in roots, leaves, stems, and siliques. The figure presents the main sucrose transporters genes expressed at all stages of development study (i.e. Fig. 1a) for each organ. Four sizes of font are used to write the name of genes and indicate their different level of expression in each organ: height (font 14), middle (font 12), low (font 10) and slight (font 8). The novel expression of sucrose transporters genes, not reported before, is mentioned in red. See for review: **AtSUC1** (At1g71880): Sivitz et al. (2007, 2008); Schmid et al. (2005); Stadler et al. (1999); Feuerstein et al. (2010); **AtSUC2** (At1g22710): Truernit and Sauer (1995); Stadler and Sauer 1996; Lalonde et al. (2003); Sauer (2007); **AtSUC3** (At2g02860): Meyer et al. (2000, 2004); **AtSUC4** (At1g09960): Schneider et al. 2012; Endler et al. (2006); **AtSUC5** (At1g71890): Baud et al. (2005); **AtSUC9** (At5g06170): Sivitz et al. (2008). **AtSWEET9** (At2g39060): Chen et al. (2012); **AtSWEET10** (At5g50790): Chen et al. (2012); **AtSWEET11** (At3g48740): Chen et al. (2012); Chen (2013); Chen et al. (2015); Le Hir et al. (2015); **AtSWEET12** (At5g23660): Chen et al. 2012; Chen (2013); Chen et al. (2015); Le Hir et al. (2015); **AtSWEET13** (At5g50800): Chen et al. (2012); Chen (2013); Chen et al. (2015); **AtSWEET15** (At5g13170): Chen et al. (2012, 2015); Seo et al. (2011); Quirino et al. (2001). The expression in roots of the five *AtSWEET* genes from the clade III: *AtSWEET 11*–*15* have been reported for the first time in our previous work (Durand et al. 2016)

The sucrose transporters (*SUC* and *SWEET*) genes were identified in the rosette, root, stem and siliques during the hydroponic development of *A. thaliana* by RT-PCR, (40 cycles) and the expression of the detected genes was specified in each organ by RT-qPCR (Fig. 10 and Table S2).

Four *AtSUC* genes *(AtSUC1*–*4*) were found expressed in rosette, roots, stem and siliques whatever the development stage considered (Fig. 10 and Table S2). Our results pointed out that in roots, the strongest expression level was measured for *AtSUC1* and in siliques, *AtSUC2* gene displayed the strongest expression whatever the organs and stages of development. We also described for the first time, the presence of *AtSUC5* and *AtSUC9* transcripts in stems while *AtSUC5* expression was found only in flowers (Baud et al. 2005) and *AtSUC9* localised in floral tissue (Sauer et al. 2004; Sivitz et al. 2007).

Concerning the *AtSWEET* genes of the clade III (Chen et al. 2012), even if the four genes *AtSWEET11*–*13* and *AtSWEET15* found expressed in leaves were already described, little was known about their expression in roots, stems and siliques. Our results confirmed the expression in root of *AtSWEET11*–*14* already identified in our previous work (Durand et al. 2016), since *AtSWEET15* expression has been already described (Seo et al. 2011), and Fig. 10 and Table S2). Although the expression level of *AtSWEET13* in roots was weak, a role in C allocation to this sink organ cannot be excluded, since it was the most expressed *AtSWEET* in roots (Fig. 10 and Table S2). The expression in this organ of *AtSWEET11*–*12* (Fig. 10 and Table S2) could be linked with expression noticed in stem secondary tissues (Le Hir et al. 2015), as in *Arabidopsis* roots, secondary tissue formation also occurs. We also identified for the first time, *AtSWEET9*–*10* and *AtSWEET13*–*14* genes in the stem, but their function in this organ remains to be studied. For *AtSWEET11*, the highest transcripts levels were noticed, followed by *AtSWEET12*–*13*, while *AtSWEET 9*–*10* and *AtSWEET14*–*15* displayed a lower expression in stems (Fig. 10 and Table S2). In siliques, our results indicated a highest expression for *AtSWEET 11*–*13* and *AtSWEET15* (Fig. 10 and Table S2) in accordance with their probable involvement in seed filling (Chen et al. 2015).

### Diel source/sink regulation: starch as the main conductor for carbon allocation?

During the vegetative phase, qualified as a phase with ‘limited trophic competition’ (Christophe et al. 2008), we first analyzed the source/sink carbon partitioning during a diel cycle (10 h light/14 h dark) on plants harvested at the (Y) stage (Fig. 1b). In *A. thaliana* rosettes, sucrose level is stable until the last point in the dark period at 9 h and the sugar peak observed in the beginning of the photoperiod (Fig. 2a) was mainly due to hexose, likely reflecting the greater growth of rosette observed at dawn (Gibon et al. 2006; Wiese et al. 2007). The progressive drop of sugar content particularly during the night (Fig. 2a) could be related to the slowdown of dark growth and to the exhaustion of starch reserves (Gibon et al. 2006; Wiese et al. 2007).

In roots, the two sugar peaks followed by a decrease (Fig. 3a) might reflect the highest growth in the first light hour, and the recovery during the night (Yazdanbakhsh et al. 2011; Stitt and Zeeman 2012). This idea is consistent with the fact that root elongation rate and root branching are related to local high sugar content (Freixes et al. 2002). At this stage of development (young, *D* + 31), we noticed that sugar level was constantly higher in roots than in leaves and, also compared to leaves, the amount of hexoses in roots remained high during the diel cycle, probably to sustain the day/nocturnal root growth phases (Yazdanbakhsh et al. 2011; Stitt and Zeeman 2012). At the end of the night, sugar content was minimal in roots as in leaves, concomitantly with the lowest leaf starch content in leaves (Fig. 3a, b). This starch pattern observed in hydroponic plants is consistent with the fact that starch accumulation and degradation are linear in leaves, with 95% of starch hydrolyzed at dawn (Smith and Stitt 2007; Graf and Smith 2011). Indeed, photosynthesis exceeds sucrose export capacity during the day, so excess carbon flows into starch buildup in the light which is then mobilized for sucrose export via the phloem at night which can equal rates of sucrose export during the day (Fondy and Geiger 1982). Diel regulation of starch turnover in leaves is important to avoid C starvation at the end of night and the link between starch degradation and its conversion to biomass led to the conclusion that starch constitutes a ‘major integrator’ of plant growth (Sulpice et al. 2009; Graf and Smith 2011; Yazdanbakhsh et al. 2011; Stitt and Zeeman 2012). This nocturnal strong degradation of starch observed in leaves was accompanied by an increase in the expression of three sucrose transporter genes, *AtSUC2*, *AtSWEET11,* and *AtSWEET12* (Fig. 2c, d). Their co-expression and their diel regulation were already suggested (Haydon et al. 2011; Chen et al. 2012), which is not surprising as they are all three implicated in phloem loading (Truernit and Sauer 1995; Chen et al. 2012). High level of expression of those three genes together with starch degradation could regulate the amount of sucrose in leaves by increasing phloem sucrose loading. Indeed, recent studies on *Arabidopsis* indicated that the up-regulation of *SUC2* resulted in an enhanced ^14^C-sucrose uptake into the veins of leaf disks, which drives a higher phloem loading, and a greater part of ^14^C being translocated to the roots (Dasgupta et al. 2014). Therefore, it is reasonable to assume that an induction in the expression of this sucrose transporter gene is associated with an increased sucrose transport activity (Lemoine et al. 1996; Vaughn et al. 2002). For others genes, however, the transcript gene abundance may not match protein production and activity levels (Walley et al. 2016). This sucrose transport toward the roots seems important to maintain root metabolism and growth, especially during the night, since *A. thaliana* root is not a storage organ according to our results (Fig. 3b) and in agreement with a previous study (Streb and Zeeman 2012).

In roots, our result indicated a higher expression of *AtSUC1* during the dark period (Fig. 3c), likely linked to its putative role in the uptake of sucrose, unloaded from the phloem, into growing root cells with no symplastic connection. This role confirms its function in roots development (Sivitz et al. 2007, 2008). The noticeable expression of *AtSUC2* in roots could be linked to its potential involvement in phloem unloading as suggested for ZmSUT1, an ortholog of AtSUC2 in *Zea mays* (Carpaneto et al. 2005). When the expression of the potato orthologous *StSUT1* was reduced, the growth of young tubers was transiently reduced, indicating also a role for SUT1 in this process (Kühn et al. 2003). This is in accordance with the high sugar level found in root growing zones (Freixes et al. 2002). Concerning *AtSWEET* genes, little is known about their expression in *A. thaliana* roots except for *AtSWEET15* already found in root lateral tip of young plants (Seo et al. 2011). In our results (Fig. 10 and Table S2), we have found *AtSWEET11*-*15* genes expressed in root as we already observed during water deficit in soil (Durand et al. 2016), *AtSWEET13* gene being the most expressed and its expression increased during the night as *AtSUC1*. Except a higher expression in leaves of the double mutant *atsweet11;12*, not much data were available so far about the expression of *AtSWEET13* especially in sink organs.

Altogether, these results provide some clues about a tight relationship between diel C partitioning, sucrose transporter regulation (*AtSUC1*–*2* and *AtSWEET 11*–*13*), and starch degradation in both source (leaves) and sink (roots) organs. All these regulations allow the metabolism of these organs to be maintained, and so was the sink strength of roots during the night. This proposal supports conclusions by Kolling et al. (2015) highlighting the control of carbon partitioning by the circadian clock.

### The vegetative growth phase: one source, one sink, and a steady state for carbon allocation?

During the vegetative stage, shoot/root biomass repartition remained stable between rosette (84–86%) and roots (14–16%) (Fig. 4), highlighting the balance between C supply (source = shoot) and C demand (sink = root). This result is consistent with values found by a predictive growth model which indicated that leaf biomass represented 88% of the total plant biomass at the end of the vegetative phase (Weraduwage et al. 2015). As predicted by a modelling approach (Christophe et al. 2008), shoot growth rate was maximal during the vegetative stage suggesting a strong C investment in production of photosynthetic leaves (Weraduwage et al. 2015).

Soluble sugars’ amount increased in leaves during this phase (Fig. 5a), especially glucose, to sustain maintenance and growth respiratory as indicated by the constant increase of rosette DW (Table S3). As senescence in *A. thaliana* starts before rosette is fully developed (Stessman et al. 2002), we cannot exclude that the slight increase in hexose content during the vegetative phase was provided by this process in the older leaves, since leaf sugars increase with leaf ageing (Quirino et al. 2001; Stessman et al. 2002). This trend was confirmed by a reduction in starch content which, nevertheless, remained, however, high in rosette during this phase (Fig. 5a). During these stages (Y and A), roots—as the only persistent sink—exhibited high and stable sugar levels (Fig. 5b) and a stronger growth rate (Table S2). The C gain in root was provided by phloem transport: 1/3 (Y) to 1/2 (A) of exported [U-^14^C] sucrose was directed to the root (Fig. 6), probably linked with the very high expression of the three sucrose transporters (Fig. 5c and Table S2) involved in sucrose loading into the phloem (Truernit and Sauer 1995; Chen et al. 2012). This strong C allocation to roots could be sustained by the strong expression of *AtSUC1* observed in roots (Fig. 5d and Table S2), with a probable role in sucrose unloading from phloem into growing root cells (Sivitz et al. 2008). Nevertheless, it cannot be excluded that part of the reduction in starch content in leaves was also caused by a greater C investment in leaf growth, since this stage is C consuming (Troughton 1956; Weraduwage et al. 2015). It can be noticed that during the vegetative stage, the same sucrose transporters genes were found highly expressed in leaves (*AtSUC2*, *AtSWEET11,* and *AtSWEET12*) and in roots (*AtSUC1* and *AtSWEET13*) as during a diel cycle (see above) and might participate to shoot/root C partitioning.

At the (IE) stage, the part of ^14^C found in root exudates decreased (4.1%) compared with the two former stages ((*Y*) = 19.8%; (*A*) = 19.3%), remained stable in roots and increased in leaves (Fig. 6) As it is assumed that growth depends on carbohydrates availability and that the onset of inflorescences formation decreases plant growth rate, the lower C allocation observed in roots might reflect the C reallocation to shoots to sustain growth of the stem that constitute a new sink (Christophe et al. 2008; El-Lithy et al. 2010). This idea could be supported by the fact that *AtSWEET11* and *AtSWEET12* expression in leaves and *AtSUC1* expression in roots felt slightly at this point (Fig. 5c, d).

During the (*Y*) and (*A*) stages, a significant part of C allocated to the roots was released to the external medium (Fig. 6). Even if root rhizodeposition is often understudied, this process includes C flux to microflora, from dead cells of root cap, mucilage secretion, and exudation of low molecular weight compounds (Farrar and Jones 2000; Farrar et al. 2003; Nguyen 2003). This C lost by roots into the soil is estimated to represent 17% of the C fixed by photosynthesis, close to our results (Fig. 6) (Farrar and Jones 2000; Farrar et al. 2003; Nguyen 2003). It has been proposed that the exudation process would increase when sugar concentration was high in roots, as observed in (*A*) stage (Figs. 5b, 6) (Fatichi et al. 2014).

### The reproductive growth stage: one source, two sinks, and a competitive state for carbon allocation?

At the reproductive phase, the plant biomass repartition presented variations between the three stages studied: (F), (SH), and (SR). This phase was characterized by many inflorescence stems and fruits (siliques) (Fig. 1a): two new C consuming sinks competing with roots for C allocation. This led to a reduced part of rosette and roots in total biomass (Fig. 4) and a strong increase in shoot/root ratio highlighting increase of aerial organs growth rate (Table S3). This was previously observed on grass plants during flower formation (Troughton 1956) and in *A. thaliana* where a carbon balance model indicated an increase in assimilate demand by reproductive organs (flowers and fruits).

### In leaf

During the reproductive phase, the part of rosette in total biomass constantly decreased in favor of stem (Fig. 4) as indicated in the carbon balance model (Christophe et al. 2008) and this trend was accompanied with the highest soluble sugar levels observed in leaves at (SR) stage (Fig. 5a). Our results also indicated a strong reduction of *AtRBCS* and *AtCAB1* genes expression during this phase (Fig. S1 and Table S4), in accordance with their repression by high sugar contents (Krapp et al. 1993). As C synthesis was probably diminished in rosette leaves, the high sugar level observed was probably the result of senescence, as leaves displayed senescence symptoms (Fig. 1a) as proposed by several authors (Quirino et al. 2001; Pourtau et al. 2006). Our results indicated a high expression of the early senescence marker *AtSAG12* (Fig. S1 and Table S4) and of *AtSWEET15* gene (Fig. 5c) (formally named *AtSAG29*) at the later stages of senescence, as previously described (Quirino et al. 1999; Seo et al. 2011). Concerning *AtSWEET11* and *AtSWEET12*, their expression was increased probably to facilitate the transport of carbon excess from leaf to the phloem towards stems and siliques. Some authors have suggested a putative role for SWEET transporters in carbon remobilization during senescence process (Seo et al. 2011), and recent work demonstrated that both AtSWEET11 and AtSWEET12 can transport glucose and fructose (Le Hir et al. 2015). Our results of ^14^C partitioning also indicated that at (SR), rosette presented a lower level of C allocated (Fig. 6) highlighting C reallocation to inflorescence with a dominant assimilate demand at this stage (Christophe et al. 2008).

At the last stage (SH) of the reproductive phase, the part of rosette biomass was the lowest (Fig. 4), soluble sugar and starch contents dropped (Fig. 5a), and symptoms of senescence became very extended on older leaves (Fig. 1a), supporting the idea that less carbon was available in the rosette. Even if leaf carbon remobilization associated with senescence is a preliminary step before floral transition, it is also known to ensure growth of reproductive organs and, finally, seed filling at the end of development (Lim et al. 2007). However, at the last stage, ^14^C partitioning to the leaves rose up indicating that stem was less favored (Fig. 6). This could be due, on one hand, to the supply of C to the stay green leaves observed on hydroponic rosettes (Fig. 1a) as hydroponic culture favors vegetative biomass accumulation (Gibeaut et al. 1997; Arteca and Arteca 2000; Norén et al. 2004). This hypothesis is consistent with the continuous high expression of sucrose transporter genes implicated in phloem loading in leaves (*AtSUC2*, A*tSWEET11,* and *AtSWEET12*) (Fig. 5c). On the other hand, the presence of source cauline leaves on stem, considered as photosynthetically active, could provide a great amount of C needed by flowers and siliques (Earley et al. 2009). This is in accordance with the high soluble sugars and starch amount measured on stem containing flowers at SR and SH (Fig. 5e), indicating that stem also had a source role at these stages. This last remark was supported by the high level of expression in stems of *AtSUC2*, *AtSWEET11,* and *AtSWEET12* (Fig. 5g), which are involved in sucrose loading into the phloem to nourish new sinks organs (Chen et al. 2012; Le Hir et al. 2015).

### In roots

Carbon investment in roots during the two reproductive phases (F and SH) is reduced when both inflorescences and rosette needed C to sustain their growth and metabolism, indicating a strong trophic competition in plant in agreement with the previous works of Weraduwage et al. (2015). This phenomenon was illustrated by a great ^14^C reallocation to stem and a high level of ^14^C export to rosette (Fig. 6) during the two first stages of this phase (F and SR). Despite reduced C allocation to roots during reproductive phase, its sugar content stayed stable (Fig. 5b), likely highlighting the continual preservation of root metabolism in particular for root respiration (Lambers et al. 1996). Moreover, at the F stage, sugar content in stems was similar to the one measured in roots (Figs. 5e, b) and could be linked with a high sink activity of stem at this stage.

At (SR) and (SH) stages, a slight increase in ^14^C partitioning to roots was noticed (Fig. 6), probably because the presence of source cauline leaves on stem provides sugars to the inflorescence stems and reproductive organs (Earley et al. 2009). This trend is correlated with the high level of transcripts noticed in stems for *AtSUC2*, *AtSWEET11,* and *AtSWEET12* (Fig. 5g). These results are in agreement with the carbon balance model proposed by (Christophe et al. 2008). These authors showed that trophic competition in plant increases at the end of inflorescences development, with a higher C allocation into the fruits that are very strong sinks. In siliques at (SH) stage, the soluble sugars’ content was very high and hexoses represented 70% of soluble sugars and sucrose 30% (Fig. 5f). High level of hexoses (fructose and glucose) was already observed in the early maturation seeds of the ecotype WS (Wassilewskija) to sustain cell division (Koch 2004) and allow storage compound (starch, proteins, and lipids) synthesis at the end of seeds maturation (Baud et al. 2008; Angeles-Núñez and Tiessen 2011). This strong C gain of seeds was supported by the expression of sucrose transporters genes as *AtSUC5* and *AtSUC3* in siliques (Fig. 5h), agreeing with their involvement in sucrose uptake into embryo (Meyer et al. 2004; Baud et al. 2005; Stadler et al. 2005). *AtSWEET11*, *AtSWEET12,* and *AtSWEET15* genes are also involved in *A. thaliana* seed filling (Chen et al. 2015), in accordance with their high expression in siliques (Fig. 5h). Even if fruits are a very strong sink strength at the (SH) stage, roots remained an active sink, while *AtSUC2* was simultaneously upregulated in roots. This might be linked to the slight increase in ^14^C partitioning to roots, since it has been proposed that, in *A. thaliana,* this transporter is important for the retrieval into the phloem of leaked sucrose along the phloem pathway (Srivastava et al. 2008; Gould et al. 2012). Thus, its up-regulation observed in roots at the stages of development may favor sucrose unloading in the release phloem to nourish cells of sink organs (Srivastava et al. 2008; Gould et al. 2012). In the same way, *AtSUC1* transcripts level was equivalent as in (*A*) stage, maybe, to support an increase in C allocation to roots in accordance with its role in sucrose unloading (Sivitz et al. 2008). *AtSUC1* and *AtSUC2* gene expression regulation could be useful to maintain and/or increase C demand in roots, since sink strength is tightly associated with the capacity to reduce photo-assimilate concentration in the release phloem sieve elements (Wardlaw 1990). As a result, this would allow phloem sap movement by maintaining sucrose gradient between leaves and roots. This proves that even if inflorescence stems containing siliques are strong sinks, root remains an active sink throughout (Christophe et al. 2008).

Finally, we showed that C partitioning during development might partly be related to sucrose transporters expression and sugar content pattern. In conclusion, ontogenic development has to be considered because of its impact on C remobilization and C supplies.

### No carbon reallocation toward roots under osmotic stress

To complete the knowledge on source–sink relationship in response to environmental changes, *A. thaliana* plants were subjected to an osmotic stress as abiotic stress is known to alter carbon allocation (Minchin et al. 1994; Muller et al. 2011; Lemoine et al. 2013).

By mimicking reduced water availability, we aimed at changing carbon shoot–root allocation and studying subsequent change in sucrose transporter genes expression. The progressive increase of PEG in the root medium was finally interpreted at the molecular level as reduced water availability by plant, as suggested by the strong reduction in *AtTIP1.2* transcripts level (Fig. 8d) and increased *AtRD29a* gene expression (Fig. 8b) in roots and shoots, respectively (Alexandersson et al. 2005; Harb et al. 2010). In addition to preventing biomass accumulation in both shoots and roots (Table 1), one of the principal effects of osmotic stress was leaf wilting which appeared in the last days of osmotic stress phase. This symptom on leaves suggests an alteration in shoot water status rather than stomatal closure (Flexas et al. 2006; Durand et al. 2016). These results indicated that osmotic stress causes more damage on hydroponic plants than water deficit applied on plants growing on soil into rhizobox (Durand et al. 2016). This could be linked to decreased radiolabelled sucrose export capacity of source leaves (Fig. 9), because sucrose translocation, according to bulk flow, requires water efflux from xylem to the phloem (Münch 1930). Diminished shoot source strength was accompanied by a strong sugar accumulation in shoot (Fig. 7a), a sugar pattern already reported in response to drought in several crop species as *Vitis vinifera* (Medici et al. 2014) and *Zea mays* (Kim et al. 2000) but also in *A. thaliana* plants (Hummel et al. 2010; Durand et al. 2016). Sugar accumulation could be explained by the uncoupling between shoot growth rate and carbon assimilation: during water deficit, leaf growth is altered earlier than photosynthesis (Muller et al. 2011). Nonetheless, the higher increase in shoot sugar content compared to several studies, and particularly glucose accumulation, underlines another reason for this sugar accumulation in plants (Hummel et al. 2010). As sugars accumulation has a marginal contribution in osmotic potential (Hummel et al. 2010; Durand et al. 2016), this sugar increase could be tightly correlated to leaf senescence in *A. thaliana* (Quirino et al. 2001), because senescence is responsive to sugars (Pourtau et al. 2006). Interestingly, *AtSAG12* expression, encoding for a cysteine protease exhibiting a strictly senescence-associated expression pattern in leaves (Gan and Amasino 1995), was highly enhanced in response to osmotic stress (Fig. 8b), suggesting that hexose accumulation induced by osmotic stress may be senescence-associated. We cannot exclude that PEG treatment applied induces also a chemical stress, while moderate water deficit does not enhance *AtSAG12* expression and plants display a delayed rather than accelerated senescence (Durand et al. 2016).

Since SUC and SWEET transporters are involved in sucrose translocation between sources and sink organs, their regulation in shoot under osmotic stress-induced senescence had to be considered. Among the genes expressed in shoots, *AtSWEET12*, *AtSWEET13,* and, more particularly, *AtSWEET15* were up regulated under osmotic stress (Fig. 8a), as seen during reproductive phase (Fig. 5c). *A. thaliana* plants overexpressing *AtSWEET15* showed accelerated senescence and salt hypersensitivity, while *atsweet15* mutant were less sensitive (Seo et al. 2011). However, translational fusion with GUS under control of its own promoter indicates that AtSWEET15 protein does not accumulate to high level during senescence (Chen et al. 2015; Eom et al. 2015). For these reasons, and taking into account the fact that *AtSWEET15* was not the only SWEET transporter gene induced under osmotic stress and during development in shoot (Figs. 8a, 5c), the role of SWEET transporters in carbon recapture during stress or senescence would need to be reevaluated in the future.

Root biomass accumulation was reduced after osmotic stress phase (Table 1), but S/R ratio was only slightly reduced, a result not expected since drought usually results in a decreased S/R ratio (Chaves et al. 2003). The slight decrease in S/R was probably due to the osmotic stress protocol, because PEG was added progressively (Fig. 1c). As a result, the time for stress setting-up was likely too short compared to the age of plants to significantly alter final biomass partitioning, a measure integrating whole-carbon allocation history. The absence of carbon reallocation suggested by marginal change in S/R ratio was confirmed by evaluating translocated radiolabelled sucrose repartition in plant, which did not exhibit any increase in roots but rather a slight decrease (Fig. 9). This result is not in accordance with *Arabidopsis* plant response during a moderate water deficit in soil that exhibited an enhanced C allocation to root (Durand et al. 2016). This discrepancy is probably linked to alteration in shoot water status of plants during an osmotic stress. Even though the amount of radiolabelled sucrose translocated to root was reduced (Fig. S2), sugars content increased (Fig. 7a), probably because C exported to root was higher than its consumption. Despite increased sugars content, several sucrose transporter genes (*AtSUC1*, *AtSUC2*, *AtSWEET11*, *AtSWEET12*, *AtSWEET13,* and *AtSWEET15*) displayed reduction in their expression.

*AtSUC1* was found expressed in the root tips (Sivitz et al. 2008). Hence, decreased *AtSUC1* expression (Fig. 8c) might be associated with the reduced root growth observed (Table 1) as also noticed during a moderate water deficit (Durand et al. 2016). This reduced expression of *AtSUC1* could occur through negative control of ABA signalling (Hoth et al. 2010), itself involved in root drought stress response (Davies and Zhang 1991). Conversely to the response of the moderate water deficit, lower transcript level of *AtSUC2* was observed during an osmotic stress. This could be related to a smaller need for sucrose retrieval along the phloem pathway and unloading, since sucrose translocated to root was reduced under osmotic stress (Fig. 9 and Asensi-Fabado et al. 2015). It has also been proposed that SWEETs might be involved in sucrose leakage along phloem transport to nourish surrounding tissues (Ludewig and Flügge 2013). Hence, *SWEET11*–*15* (excepted *AtSWEET14*) downregulation in root in response to osmotic stress (Fig. 8c) might be linked to the limited need for sucrose leakage to maintain phloem sap flux down to the release phloem. This result confirms the opposite response between osmotic stress and moderate deficit water where all *SWEET11*–*15* were induced (Durand et al. 2016).

To check that physiological effects and variations of sugar contents and sucrose transporters gene expression were caused by PEG addition in the nutrient medium, the experiment was extended one additional week, during which PEG has been removed from the stressed plant nutrient medium. Growth recovery was partial in shoot, since rosette growth was still lower than control plants at the end of rewatering phase (Table 1). Conversely, root growth during the rewatering phase was still decreasing compared to plants at the end of stress, probably due to the loss of root integrity after their contact with the PEG solution. This trend was also observed concerning sugar content, because we measured higher sugar contents in leaves in stressed plants after rewatering, even though osmotic potential and water content had been restored (Table 1). Marker gene expression measurements confirmed that this pattern was especially due to PEG, since it vanished after rewatering phase (Fig. 8b, d). Concerning shoot and root sucrose transporter genes, we also confirmed a global recovery in their expression, confirming that modifications previously noted were the consequence of PEG addition in the nutrient medium. It has to be pointed out that some SWEET transporter genes even experienced an up-regulation in shoot (*AtSWEET15*) and in roots (*AtSWEET 11, 12,* and *15*) after the rewatering phase, and could be linked to the increase in radiolabelled sucrose translocation toward root and shoot (Fig. S2). Finally, we confirmed that the effects observed were due to PEG addition, even though plant recovery was incomplete for some parameters. Nonetheless, this tends to show that osmotic stress applied was partially reversible.

In this work, we provide evidence for a transcriptional regulation of root sucrose transporters expression in response to osmotic stress (Fig. 8c) and we showed a tight relationship between decreased gene expression of several sucrose transporters and reduction in amount of radiolabelled sucrose translocated towards root. According to the “pull hypothesis” of shoot–root interaction (Farrar and Jones 2000), downregulation of *AtSUC2* and several *AtSWEET* genes in osmotic stressed roots (Fig. 8c) could contribute to alter the sucrose flux from the shoot to the root release phloem.

## Conclusion

Taking advantage of the hydroponic culture system allowing efficient growth of plants up to seeds harvest and the collection of the entire root system, the study of source-to-sink carbon allocation in relation to sucrose transporter gene expression was investigated. To get a large overview of contrasted source/sink relationships, this study was followed at two time scales: during a day/night cycle or during the full life cycle of plants and at a characteristic environmental scenario corresponding to an abiotic (osmotic) stress (progressive addition of PEG) applied on plants.

In roots, the high expression of specific sucrose transporters genes (*AtSUC1*–*2* and to a lesser extent *AtSWEET11*–*13*) specify their role in C allocation to roots in accordance with Durand et al. (2016). In the aerial organs, the strong expression of *AtSUC2* and *AtSWEET11*–*12* confirms their implication in sucrose phloem traffic (loading and retrieval). Sucrose level was highly stable in roots and leaves whatever the time scale considered. Total sugar variation in both organs was mainly due to accumulation of hexoses especially glucose, probably sustained by starch consumption.

Circadian regulation of carbon allocation between rosette and roots and steady-state level of sucrose in both organs were linked with C metabolism, mostly diel starch synthesis and degradation. Among the whole gene family of sucrose transporter tested, only a subset of SUC and SWEET transporters were expressed. Their expression level did not show dramatic changes during this period excepted during the night when the expression of *AtSUC2* (sucrose loading in phloem) in leaves and *AtSUC1* (sucrose unloading) in roots increased and starch degradation was higher. This is in accordance with a continuous flow of sugar during the day (sucrose derived from photosynthesis) and during the night (sucrose derived from starch degradation). Therefore, the time for harvest in subsequent experiments was set to 1300 h.

On a longer time scale (full development), biomass repartition between rosette (one source) and roots (one sink) indicated a steady state at the vegetative stage finely regulated by stable sucrose level in both organs and a balanced C allocation between leaves and roots. Nonetheless, there was a switch in C allocation between the vegetative (sinks limited to young leaves and roots) and the reproductive stage with two new sinks studied: stem and siliques. Because of the competition of these new sinks, roots represented a decreasing part of the total biomass, with a significant drop between the Flowering (F) and the Silique Ripening (SR) stages. This may be related to the decrease of carbon transport from rosettes (source) to the roots (sink) (from the ^14^C-sucrose transport experiments) at the earlier stage (Inflorescence to Flowering). In the rosette, sucrose level remained stable even if glucose is highly accumulated and a constant increase in the expression of genes of transporters implicated in sucrose phloem loading (*AtSUC2* and *AtSWEET11*–12) was observed. This could indicate a strong regulation of phloem sap content to equilibrate sucrose gradient between leaves and both sinks organs (roots and stems) and reflects a finely tuned C allocation between those organs. In the roots, the major difference was the increase of *SUC2* expression and C allocation at the latter stages of development. This result could be related to the higher competition between sinks (stem and siliques versus roots) and highlights the importance to maintain a functional root system until the full development of seeds. During osmotic stress, roots growth was reduced and ^14^C transport was diminished concomitantly with the inhibition of the expression of the main sucrose transporter genes found in roots (*AtSUC1, AtSUC2,* and *AtSWEET11*–*13 and 15*) and sugar content. Those trends indicate a strong alteration of C allocation in osmotic stressed plants, not observed during a moderate water deficit (Durand et al. 2016). This obviously indicated two different ways to cope with drought stress, while PEG osmotic stress is associated with damage, which accelerated senescence and sucrose transporters repression reflecting a drastic impact of this stress on plants probably linked with a chemical impact of the PEG solution. Altogether these data indicate a major link for a subset of transporters and sucrose import in the roots and thus in root sink strength, whatever the condition tested (24 h cycle, full life cycle development and osmotic stress).

### *Author contribution statement*

MD performed most the experiments, conceived experiments and research plans, analyzed the data, and wrote a part of the article. DM performed experiments, conceived experiments and research plans, and analyzed the data. This article is part of the MD’s and DM’s PhDs work. BP provided technical support. LM performed the transport of [U-^14^C] sucrose experiments and data analyses. RL supervised the experiments, conceived research plans, and complemented writing. NP conceived experiments, supervised experiments and research plans, and wrote most of the article. MD, LM, RL, and NP discussed the data and revised the article. All the authors have read and approved the manuscript.

## Electronic supplementary material

Below is the link to the electronic supplementary material.

Supplementary material 1 (PDF 325 kb)

Supplementary material 2 (DOCX 34 kb)

## Abbreviations

A: Adult stage
Ctrl: Control plant
F: Flower stage
IE: Inflorescence emergence stage
SH: Silique harvest stage
SR: Silique ripening stage
St: Stressed plant
Y: Young stage
