# Supplementary material for: Carbon source–sink relationship in Arabidopsis thaliana: the role of sucrose transporters
Source: Planta. 2017 Nov 14;247(3):587–611. doi: 10.1007/s00425-017-2807-4 (PMC5809531; doi:10.1007/s00425-017-2807-4)
Supplement: Supplementary file 1 — Supplementary material 1 (PDF 325 kb) [file 425_2017_2807_MOESM1_ESM.pdf]

Figure S1

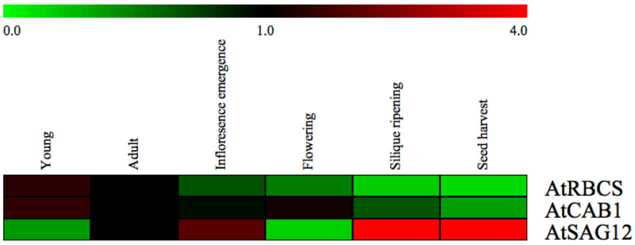

0.0
1.0
4.0
Young
Adult
Infloresence emergence
Flowering
Silique ripening
Seed harvest
AtRBCS
AtCAB1
AtSAG12

Figure S2

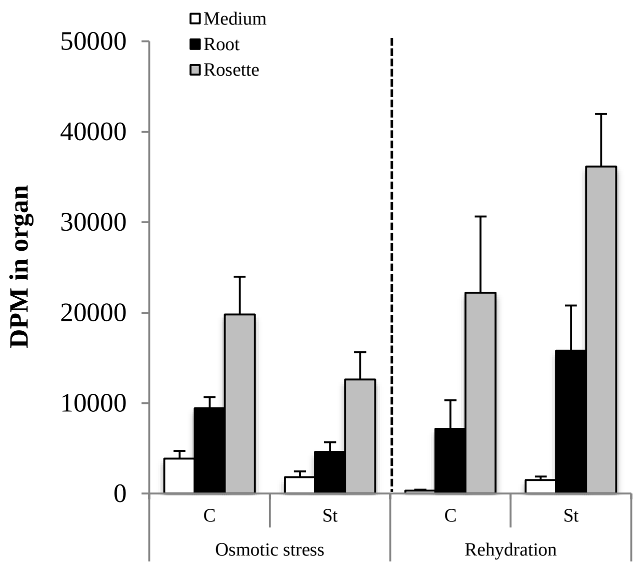
Medium
Root
Rosette
50000
40000
30000
20000
10000
0
DPM in organ
C
St
C
St
Osmotic stress
Rehydration

Table S1. Primers used to study the expression pattern of the corresponding genes. Nine *AtSUC* genes: *AtSUC1* (*At1g71880*), *AtSUC2* (*At1g22710*), *AtSUC3* (*At2g02860*), *AtSUC4* (*At1g09960*), *AtSUC5* (*At1g71890*), *AtSUC6* (*At5g43610*), *AtSUC7* (*At1g66570*), *AtSUC8* (*At2g14670*), *ATSUC9* (*At5g06170*) and 7 genes of *AtSWEET* (clade III): *AtSWEET9* (*At2G39060*), *AtSWEET10* (*At5g50790*), *AtSWEET11* (*At3g48740*), *AtSWEET12* (*At3g23660*), *AtSWEET13* (*At5g50800*), *AtSWEET 14* (*At4g25010*), *AtSWEET15* (*At5g13170*). Osmotic stress markers genes: *AtRD29a* (*At5G52310*) and *AtTIP1.2* (*At3G26520*). Photosynthesis genes: *AtRBCS* (*At1G67090*) and *AtCAB1* (*At1G29930*). Senescence marker gene: *AtSAG12* (*At5G13170*)

Genes	Accessions	Forward primers	Reverse primers
AtSUC1	AT1G71880	GACCTTTCGACGCCTTGTTC	AATACTCCACTAATCGCCGCTG
AtSUC2	AT1G22710	GGTAAGTGGTGTATTGGCGTTG	GAGCCAAACAACCACTGCTAAA
AtSUC3	AT2G02860	CAAGAACCGCAGCCGTAATC	CTTGACCGCCACCGGAAT
AtSUC4	AT1G09960	AGTGTCAAGCGAGGAACGCATA	AGTCACACGAGAAGCCATTGC
AtSUC5	AT1G71890	GGGCTATGGGATTCCATTAG	TAAAAGACAGACGACCAAGG
AtSUC6	ATG543610	TCCTGTCTCCGGCCTGCTT	AGGCGCCCATAGCGATGA
AtSUC7	AT1G66570	GTCTTTAAGAGACAAGCCCAC	AGACTGTCTATCCACAGTCGT
AtSUC8	AT2G14670	CTAGCTTCCATAATCTCAAGT	TTGGTAAGTTTCCACCTCCAAAA
AtSUC9	AT5G06170	GTGGTTCCTGATGAGCCG	GAGAAGCTGAACGTATGGG
AtSWEET9	AT2G39060	ACGCCGTCATGTGGTTCTTT	TGCTAGTTGGTTCTCTGTTGGC
AtSWEET10	AT5G50790	AACTCCTTTGCCTTTGTCGTACA	GTCAACACGAAGATTGCGCC
AtSWEET11	AT3G48740	TCCTTCTCCTAACAACTTATATACCATG	TCCTATAGAACGTTGGCACAGGA
AtSWEET12	AT5G23660	AAAGCTGATATCTTTCTTACTACTTCGAA	CTTACAAATCCTATAGAACGTTGGCAC
AtSWEET13	AT5G50800	CTTCTACGTTGCCCTTCCAAATG	CTTTGTTTCTGGACATCCTTGTTGA
AtSWEET14	AT4G25010	AAACGCTGTGGGATGCTTCA	TTCAAGAGCCCAAGAACCTTCA
AtSWEET15	AT5G13170	CAATGACATATGCATAGCGATTCCAA	GGACTCATCACGACAATACTCTTAAG
AtSAG12	AT5G45890	TGGATACGGCGAATCTACTAACG	GCTTTCATGGCAAGACCACATAG
AtRD29a	AT5G52310	GAAAGGAGGAGGAGGAATGGTT	GGAGCCAAGTGATTGTGGAGA
AtRBCS	AT1G67090	ACGGTACTGGACAATGTGG	CGAATCCGATGATCCTAATG
AtCAB1	AT1G29930	CCAGAGGCATTCGCTGAGTTG	CCTTACCAGTGACGATGGCTTG
AtTIP1.2	AT3G26520	CAAGAACGGTAGTCTCGGAACA	GACCAGCCCAGTAAACCCAGT
Reference	AT5G12240	AATATCGCTTTGCAGCTTCTG	GATTTTGCAAGGCTTTCGAG

Table S2 *AtSUC* and *AtSWEET* gene expression in different organs of plants for the six principal growth stages of *A. thaliana* grown hydroponically. Relative expression of *AtSUCS* and *AtSWEET* is measured by RT-qPCR. Data are expressed as normalized expression (no unit) to the reference gene *At5g12240* expression level (Czechowski et al. 2005) and are obtained from the pool of plants ($n \geq 12$) of 11 independent experiments

Development stage

	Young		Adult		Infloresence emergence		Flowering			Silique ripening				Seed harvest			
	Root	Rosette	Root	Rosette	Root	Rosette	Root	Rosette	Stem	Root	Rosette	Stem	Silique	Root	Rosette	Stem	Silique
AtSUC1	4.170	1.966	2.445	1.133	1.932	2.014	1.959	3.010	2.313	2.908	2.621	3.204	1.035	3.138	2.603	4.925	0.853
AtSUC2	0.847	6.000	0.616	3.630	0.753	3.945	0.901	4.757	4.691	1.376	3.918	7.111	16.223	1.301	3.182	9.918	12.641
AtSUC3	0.514	0.642	0.257	0.390	0.287	0.429	0.349	0.470	0.463	0.349	0.555	0.514	0.403	0.314	0.460	0.753	0.145
AtSUC4	0.240	0.123	0.149	0.091	0.140	0.104	0.171	0.112	0.104	0.179	0.120	0.120	0.403	0.157	0.132	0.154	0.173
AtSUC5	ND	ND	ND	ND	ND	ND	ND	ND	0.101	ND	ND	0.084	2.676	ND	ND	0.089	2.329
AtSUC6	ND	ND	ND	ND	ND	ND	ND	ND	ND	ND	ND	ND	ND	ND	ND	ND	ND
AtSUC7	ND	ND	ND	ND	ND	ND	ND	ND	ND	ND	ND	ND	ND	ND	ND	ND	ND
AtSUC8	ND	ND	ND	ND	ND	ND	ND	ND	ND	ND	ND	ND	ND	ND	ND	ND	ND
AtSUC9	ND	ND	ND	ND	ND	ND	ND	ND	ND	ND	ND	0.001	ND	ND	ND	0.001	ND
AtSWEET9	ND	ND	ND	ND	ND	ND	ND	ND	ND	ND	ND	0.304	ND	ND	ND	0.057	0.063
AtSWEET10	ND	ND	ND	ND	ND	ND	ND	ND	0.018	ND	ND	0.043	0.697	ND	ND	0.030	0.020
AtSWEET11	0.012	1.670	0.032	0.801	0.006	0.660	0.010	1.310	0.241	ND	1.329	0.966	3.681	ND	1.790	1.283	0.227
AtSWEET12	0.004	0.082	0.060	0.117	0.040	0.092	0.043	0.259	0.063	0.014	0.356	0.275	0.774	0.021	0.500	0.218	0.107
AtSWEET13	0.118	0.318	0.068	0.155	0.069	0.157	0.079	0.164	0.071	0.057	0.157	0.354	0.444	0.040	0.137	0.451	0.366
AtSWEET14	0.025	ND	0.009	ND	0.008	ND	0.005	ND	0.007	0.003	ND	0.062	0.042	0.005	ND	0.039	0.013
AtSWEET15	0.003	0.004	0.002	0.050	0.002	0.080	0.001	0.028	0.009	0.002	0.768	0.232	0.232	0.003	1.231	0.235	0.235

Table S3 Growth parameters of *A. thaliana* (cv Columbia) grown hydroponically. Dry weight (DW) measurements on roots, rosette and stem at six principal growth stages Shoot/Root ratio is calculated from DW of the same samples. Data are expressed as the mean ± SE of seven to eight individual plants from two separated experiments. (-) organ not present

	Plant stage development					
	**Young**	**Adult**	**Inflorescence emergence**	**Flowering**	**Silique ripening**	**Seed harvest**
Root DW (mg)	4.1 (0.3)	41.4 (3.6)	108.9 (8.6)	205.4 (23.1)	219 (26.4)	376.6 (24)
Rosette DW (mg)	22.2 (1.7)	232.6 (17.1)	693.7 (61)	1274.6 (97.6)	1511.3 (200.8)	1626.6 (146.4)
Stem DW (mg)	-	-	2.3 (0.5)	193.9 (44.9)	1008.7 (93)	3515.1 (742.4)
Shoot:Root DW	5.5 (0.4)	5.7 (0.2)	6.4 (0.2)	7.3 (0.3)	12 (0.8)	13.3 (1.3)

Table S4 Expression of *AtSAG12*, *AtRBCS* and *AtCAB1* genes in leaves at the 6 different growth stages. Relative expression is measured by RT-qPCR. Data are expressed as normalized expression (no unit) to the reference gene *At5g12240* expression level (Czechowski et al. 2005) and are obtained from the pool of plants ($n \geq 12$) of 11 independent experiments

	**Young**	**Adult**	**Infloresence emergence**	**Flowering**	**Silique ripening**	**Seed harvest**
AtRBCS	4803.93	3147.52	2134.97	1584.71	625.99	415.87
AtCAB1	1552.09	989.12	935.76	1192.69	661.68	372.22
AtSAG12	0.07	0.16	0.34	0.03	1.04	0.90
