# Supplementary material for: Carbon source–sink relationship in Arabidopsis thaliana: the role of sucrose transporters
Source: Planta. 2017 Nov 14;247(3):587–611. doi: 10.1007/s00425-017-2807-4 (PMC5809531; doi:10.1007/s00425-017-2807-4)
Supplement: Supplementary file 2 — Supplementary material 2 (DOCX 34 kb) [file 425_2017_2807_MOESM2_ESM.docx]

**Legend of supplementary Figures**

**Fig.S1** Evolution of fold change of *AtRBCs, AtCAB1* and *AtSAG12* gene expression in leaves and at the six principal growth stages of *A. thaliana* grown hydroponically. Fold change of expressed *AtRBCs, AtCAB1* and *AtSAG12* genes in rosette. Fold change values are displayed as a two colours heat-map view, with rows corresponding to the genes of interest and columns to the 6 development stages (MeVsoftware (<http://www.tm4.org/mev.html>). The primers used in RT-qPCR experiments are presented in the Table S1). Fold change values are obtained by comparison with the adult stage after normalization to the reference gene At5g12240, (Czechowski et al. 2005)). Data are the mean of measures obtained from pools of five plants

**Fig.S2** Quantitative representation of [U-^14^C] sucrose transport (expressed as DPM of total radioactivity exported) from a mature leaf to sink leaves, roots and external medium. For each condition studied (osmotic and rewatering phases), plants are fed with a drop (10 µl) of ^14^C sucrose on a mature leaf, after gentle scrubbing with carborundum. After 5 h of transport, the radioactivity is counted in the rosette, roots and external medium. Each result is the mean ± SE of measures obtained from three to eight individual plants from two separated experiments
